# Spatial dynamics of synthetic microbial mutualists and their parasites

**DOI:** 10.1371/journal.pcbi.1005689

**Published:** 2017-08-21

**Authors:** Daniel R. Amor, Raúl Montañez, Salva Duran-Nebreda, Ricard Solé

**Affiliations:** 1 Physics of Living Systems, Department of Physics, Massachusetts Institute of Technology, Cambridge, Massachusetts, United States of America; 2 ICREA-Complex Systems Lab, Department of Experimental and Health Sciences, Universitat Pompeu Fabra, Barcelona, Spain; 3 Institute of Evolutionary Biology (CSIC-Universitat Pompeu Fabra), Barcelona, Spain; 4 Centre for Biomedical Network Research on Rare Diseases (ISCIII), Málaga, Spain; 5 Santa Fe Institute, Santa Fe, New Mexico, United States of America; University of Chicago, UNITED STATES

## Abstract

A major force contributing to the emergence of novelty in nature is the presence of cooperative interactions, where two or more components of a system act in synergy, sometimes leading to higher-order, emergent phenomena. Within molecular evolution, the so called *hypercycle* defines the simplest model of an autocatalytic cycle, providing major theoretical insights on the evolution of cooperation in the early biosphere. These closed cooperative loops have also inspired our understanding of how catalytic loops appear in ecological systems. In both cases, hypercycle and ecological cooperative loops, the role played by space seems to be crucial for their stability and resilience against parasites. However, it is difficult to test these ideas in natural ecosystems, where time and spatial scales introduce considerable limitations. Here, we use engineered bacteria as a model system to a variety of environmental scenarios identifying trends that transcend the specific model system, such an enhanced genetic diversity in environments requiring mutualistic interactions. Interestingly, we show that improved environments can slow down mutualistic range expansions as a result of genetic drift effects preceding local resource depletion. Moreover, we show that a parasitic strain is excluded from the population during range expansions (which acknowledges a classical prediction). Nevertheless, environmental deterioration can reshape population interactions, this same strain becoming part of a three-species mutualistic web in scenarios in which the two-strain mutualism becomes non functional. The evolutionary and ecological implications for the design of synthetic ecosystems are outlined.

## Author summary

In order to achieve greater levels of complexity, complex systems often display cooperative interactions that enable the formation and stabilisation of mutualisms. Theoretical models have shown that closed chains of cooperative species or *hypercycles* might have been crucial in the evolution towards complexity in early molecular replicators. However, parasites can easily destroy the cooperative loop, unless the system is embedded in a spatial context where interactions are limited to nearest neighbours. A dynamically similar phenomenon occurs in ecological webs, where closed positive feedback loops contribute to global stability and ecophysiology. Here we explore this problem by engineering synthetic cooperative strains of microbes that grow and interact in a cell culture under the absence and presence of a synthetic parasitic strains. By analysing the impact of cooperation under different conditions, we find that cooperative replication is successful and overcomes competitive interactions in nutrient-poor environments. However, the same closed loop fails to establish in nutrient-rich media. Moreover, parasitic entities that jeopardise cooperation under well-mixed conditions can be overcome by hypercycles when growing in a two-dimensional space.

## Introduction

The evolution of complexity is largely grounded in the emergence of new forms of cooperation capable of holding together higher-order entities from simpler ones. Cooperative interactions have played a great role in the so-called *major transitions in evolution* [1]. Cooperation pervades the rise of molecular systems capable of overcoming mutation thresholds, multicellular assemblies incorporating division of labour or the appearance of insect societies. Each of these structures incorporates new properties that cannot be observed at the level of its component parts. Despite the burden involved in sustaining the new, larger entity, the advantage of staying together can overcome, under some circumstances, the cost of the association.

**Fig 1:**
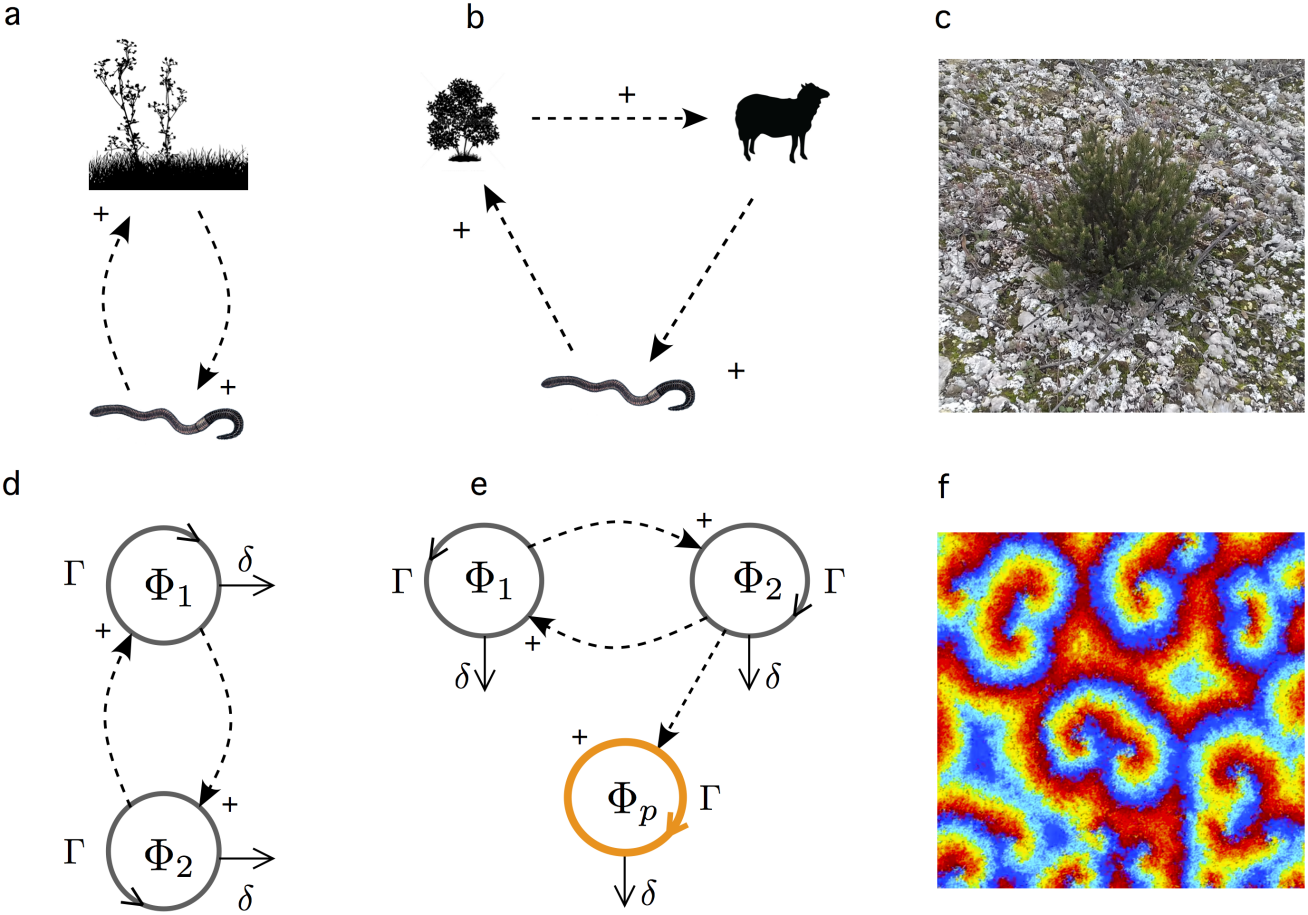
Natural and synthetic cooperative loops and their parasites. Cooperative feedback loops are widespread in ecological systems, and three examples are shown in (a-c). Here we indicate in (a) the mutual support between vegetation (grasses) and earthworms and in (b) a more complex cycle composed by vegetation, cattle and earth worms (and other invertebrates). In (c) the image shows a small area within a semiarid ecosystem including a plant surrounded by biological soil crust. Formal models of these types of interactions are described closed feedback interactions. In (d) we display the basic logical scheme of interactions for a two-component cooperative loop (a two-member hypercycle in the molecular replicators literature). In (e) we show an extended model where a parasitic species (colour circle) takes advantage of one of the species but gives no mutual feedback. In models of molecular replicators, it has been shown that parasites can easily damage cooperation, but this effect is reduced or suppressed under the presence of oscillations and spatial diffusion when spiral waves get formed (f). Here different colours indicate different molecular species in a *n* = 8 member hypercycle. In this paper we examine the role played by space and parasites in synthetic ecosystems.

Cooperation can be achieved in particular by means of closed catalytic loops. Mutualistic interactions pervade ecological communities at many different scales, from bacterial communities to microbiomes and large-scale ecosystems [2]. The presence of these reciprocal relations was already outlined by Charles Darwin in one of his memorable studies on the ecology of earthworms [3, 4] and summarised by the diagram of Fig 1a. Earthworms improve soil porosity and organic content that helps plants to grow, which results in more organic matter and mechanisms of soil preservation (which favours the earthworm population). This is a simple, two-component (*n* = 2) diagram, but ecosystems are characterised by the presence of multiple feedback loops and thus interactions might be more complex, like the three-member (*n* = 3) loop shown in (Fig 1b). Here vegetation is grazed by animals, whose activity enhances the survival of invertebrates, which in turn improve soil quality thus favouring plant growth. Because of their ecological and evolutionary relevance, cooperative interactions have also been a major topic in synthetic biology [5–9]. The possibility of engineering *de novo* cooperative loops is of relevance for several reasons. On one hand, engineered mutualisms could be used to build desirable (even optimal) functionalities that require the presence of a tight metabolic dependence [10, 11]. Moreover, the possibility of designing mutualistic interactions and even symbiotic pairs [11–15] provides a unique opportunity for exploring the emergence of cooperation in evolution under a ‘synthetic” perspective [16].

Mutualistic interactions are also required to sustain stable communities, particularly when harsh conditions are present. An example (Fig 1c) is provided by drylands [17] and in particular the interactions between the so-called biological soil crust (BSC) and vascular plants [18]. The BSC defines in itself a complex ecosystem enclosed within a few centimetres of the topsoil, largely controlling the energy and matter flow through the soil surface, helping vegetation thrive under semiarid conditions. The soil microbiome plays a major role in sustaining plant diversity and its dynamics, with the latter often completely dependent on their microbial symbionts [19]. Since these ecosystems might experience sudden declines due to climate change [20, 21] understanding their dynamics is crucial to predicting their future. In this context, it has been suggested that engineering new synthetic mutualistic loops in endangered ecosystems could help prevent catastrophic shifts [22, 23].

Understanding cooperation, its rise and fall and how can it overcome competitive interactions is an important problem. A great insight has been obtained from both field and theoretical studies [2]. An elegant description of this class of cooperative loops is the *hypercycle*, first suggested within the context of prebiotic evolution [24–28]. Here a simple catalytic system is defined (as in Fig 1a and 1b) forming a closed graph where the replication of each component is catalysed by a previous one in the loop, while it also catalyses the replication of the next. The simplest case is the one shown in Fig 1d for a two-member system [24, 29]. If we indicate by Φ_1_ and Φ_2_ their population sizes, a pair of coupled equations allows us to represent the hypercycle model as follows:

$$\begin{array}{c} \begin{array}{c} \frac{d\Phi_{1}}{dt}=\alpha_{12}\Phi_{1}\Phi_{2}(1-\frac{\Phi_{1}+\Phi_{2}}{K})-\delta_{1}\Phi_{1}\\ \frac{d\Phi_{2}}{dt}=\alpha_{21}\Phi_{1}\Phi_{2}(1-\frac{\Phi_{1}+\Phi_{2}}{K})-\delta_{2}\Phi_{2} \end{array} \end{array}$$ (1)

where *α*_*ij*_ (*i* ∈ [1, 2], *j* ∈ [1, 2]) stand for the replication rates of the cross-catalytic loop, *δ*_*i*_ is the degradation (death) rate of species *i*, and the carrying capacity *K* takes into account saturation effects that confine the hyperbolic reaction kinetics to relatively low (or moderate) population densities [30]. As defined, we can see that no proliferation of any of the two partners will occur in the absence of the other, as a consequence of the second-order kinetics that requires the product of the two concentrations.

The hypercycle can outcompete other non-cooperative species [24, 26] but a major drawback is that it can also be easily threatened by a parasite (Fig 1e) capable of destabilising the whole system [31]. Interestingly, mathematical and computer models indicate that this problem can be limited by the presence of diffusion in a spatial domain [32–35]. Hypercycles displaying spatial structures (Fig 1f) are obtained from *n* > 4 loops capable of exhibiting oscillations. In a nutshell, the spatial structure imposes a limitation to the spread of the parasite, and it can even go extinct if the inaccessibility of its target species, combined with its death rate, makes it non-viable [36].

Since mutualistic interactions are widespread in ecological networks, and the role of both space and parasites is known to be essential to sustain diversity and enhance ecosystem function, we can ask whether the concepts above can be used to study ecological interactions. The answer is yes, but needs some important clarification. As discussed in [37] we should be careful in using the label “hypercycle” to describe all types of mutualistic interactions sharing the presence of second-order terms as those described by the previous equations. We made this distinction since we will apply this class of model framework to synthetic ecosystems, which formally share this class of kinetic description but are not based on cross-catalytic replication. However, since all these model systems do share a common mathematical structure, we should expect to observe similar dynamical behaviours when space or parasites are introduced. In fact, living organisms may impose particular constraints that are classically not acknowledged in hypercycle theories. For example, physical features such as cell shape can critically influence the spatial structure of microbial populations [38], and even determine which species will survive in a given community [39]. Here, we propose engineered microbial ecosystems as an experimental system where some predictions from hypercycle-related models can be tested. In this context, recent studies involving engineered microbial mutualists have described that mutualism enhances species intermixing [40], while genetic drift [41, 42] acts against this effect during range expansions [43]. Moreover, microbial mutualists can exhibit spatial self-organization that disfavours parasites when growing into open space [44, 45]. Nevertheless, the number of studies focusing on the spatial dynamics of microbial mutualists is very limited, and determining to what extent these results are universal and which features are associated to the specific experimental system remains as an open problem.

In this paper, we address this problem by studying how engineered bacterial mutualists expand in different environments. A minimal two-member cooperative loop model provides qualitative understanding on how the mutualists transit from an obligate mutualism (dominated by hyperbolic growth) to a competition scenario (governed by Malthusian growth) as the environment becomes richer in growth-limiting resources. Surprisingly, we find that the range expansion process can be slowed down in richer environments, a feature that is associated to enhanced genetic drift effects preceding local resource depletion. Moreover, we show that a parasite strain can threaten the synthetic mutualistic community in well-mixed populations, and that environmental conditions can determine the fate of the parasite during range expansions. While the parasite is excluded from the expanding population in environments where the two-strain mutualistic loop can succeed, environmental deterioration (e.g. associated to a toxic molecule) can reshape the species interactions leading to an advancing population that necessarily includes the three strains.

## Results

### Environmental conditions modulate synthetic mutualistic interactions

**Fig 2:**
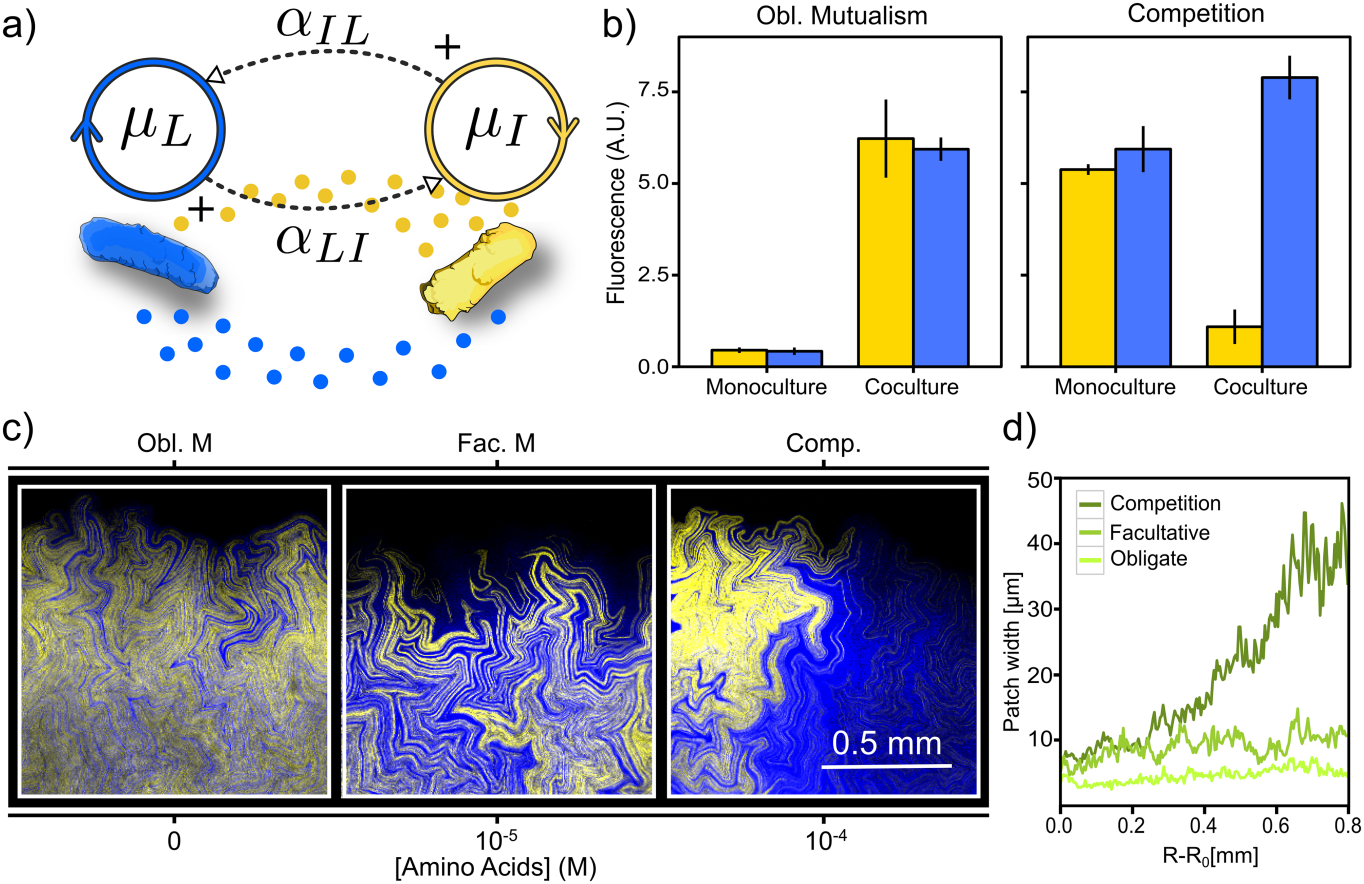
Resource availability alters interactions between synthetic mutualists and modulate genetic diversity during range expansions. *a)* We use a pair of engineered bacterial strains (yellow depicts *I ^-^* cells and blue stands for *L^-^*) that engage in mutualistic interactions by cross-feeding amino acids. *b)* Both strains are able to grow in liquid cocultures lacking both amino acids, but monocultures exhibit no growth in this conditions (Obl. Mutualism). When amino acids are supplemented at 10^−4^M (Competition), monocultures grow to comparable levels while the *L^-^* strain overcomes its partner in cocultures. Error bars show the standard deviation across 9 replicates. *c)* Bacterial mutualists develop single-strain patches during range expansions, whose spatial structure is influenced by environmental conditions (concentration of supplemented amino acids), see also S3 Fig. *d)* Width of single-strain sectors as the range expansion takes place. Obligate mutualism and facultative mutualism scenarios correspond to environments supplemented with 0 and 10^−5^ *μ*M of *iso* and *leu*, respectively, both leaving an approximately constant patch width. In contrast, the competition scenario (10^−4^ *μ*M of *iso* and *leu*) leads to an increasing patch width as the range expansion progresses. Curves show the patch width for single colonies, see replicates in S3 Fig.

Our model system for studying mutualistic interactions is composed of a pair of bacterial strains engineered to exchange essential amino acids (Fig 2a). The *I ^-^* strain (depicted in yellow) cannot produce the isoleucine (*iso*) amino acid but overproduces and leaks leucine (*leu*), while *L^-^* (in blue) cannot produce *leu* but overproduces and leaks *iso* [6]. Therefore, the strains are able to engage in a cross-feeding mutualism that permits growth in coculture, in a minimal medium lacking both amino acids where neither *I ^-^* nor *L^-^* can grow in monoculture (obligate mutualism scenario in Fig 2b). However, both *I ^-^* and *L^-^* are able to grow in monoculture when this same medium is supplemented with 10^−4^M of both *iso* and *leu*. Under these conditions, the dominant interaction between *I ^-^* and *L^-^* cells in coculture is competition for additional resources (competition scenario in Fig 2b, see also S1 and S2 Figs).

In order to further characterize the dynamics of our synthetic mutualistic system, we seeded the cross-feeding strains on agar plates with different concentrations of *iso* and *leu*. Fig 2c shows the spatial structure close to the edge of the population front after 4 days of incubation (see S3 Fig). When no amino acids are supplemented into the medium, cells are only able to grow if mutualistic partners remain close enough. The population engages in an obligate mutualism, which leads to a self-organized distribution with a characteristic high intermixing of the two strains. This high genetic intermixing of the obligate mutualists leads to relatively thin single-strain patches, whose avarage size remains approximately constant as the range expansion takes place, as shown in Fig 2d. In contrast, the competition scenario reveals a remarkably different spatial structure. When amino acids are supplemented at 10^−4^ *μ*M, the driving interaction is competition for space and resources, since cells no longer need their mutualistic partners in order to obtain the amino acids required to grow. The range expansion dynamics is thus governed by genetic drift [41], which leads to demixing of the population into progressively wider (single-strain) patches.

In between of the above two modes of invasion, we found the environmental conditions that allow a facultative mutualistic behaviour. Single-strain patches are wider than those observed in the absence of supplemented *iso* and *leu*, although genetic diversity is still preserved (patch width remains approximately constant) as the front propagates, Fig 2c and 2d. In other words, in the facultative scenario, the concentration of amino acids added to the media permit the strains to grow into wider patches (compared to those of obligate mutualists), but both strains still benefit from the cross-feeding. It is worth noting that, while (both obligate and facultative) mutualism scenarios lead to stable coexistence at the front, the competition scenario would lead to the exclusion of one of the strains at larger timescales.

Results in Fig 2 show that environmental conditions can modulate the interactions between the mutualistic species, which can lead to different dynamics during range expansions. The scenarios in Fig 2c (see also S3 Fig) reveal a qualitatively identical interplay between mutualism and genetic drift in range expansions of yeast populations Ref. [40, 43]. Even though different systems exhibit specific traits that depend on their model organisms (such as the fractal dimension of the boundary domains [38], see S5 Fig), the qualitative agreement between the results in Fig 2 and those in Refs. [40, 43] suggests an inherent dynamics of mutualism to some extent independent of the mutualistic agents.

### Slowdown of mutualistic front speed under local resource depletion in moderately rich environments

How the speed of mutualistic range expansions is affected by the environment? To approach this problem, let us first modify the minimal model [Eq. set (1)], in order to be able to describe the population expansion as a propagating front (a phenomenon that is widely used to model biological range expansions such as those of genes [46], microbial populations [47], cooperators [48] and even cultural invasions [49]). Moreover, given that single-strain cultures grow in amino acid rich environments (Fig 2), we consider amino acid supplementation as a way to introduce Malthusian growth rates in the system (as done in Ref. [9] for mutualistic yeast strains). Thus, our minimal Reaction-Diffusion (RD) model describing the spatiotemporal dynamics of the synthetic mutualistic replicators reads (see Methods):

$$\begin{array}{c} \begin{array}{c} \frac{\partial I}{\partial t}=D\frac{\partial^{2}I}{\partial r^{\;2}}+\left(\mu_{I}I+\alpha_{IL}IL\right)(1-\frac{I+L}{k}),\\ \frac{\partial L}{\partial t}=D\frac{\partial^{2}L}{\partial r^{\;2}}+\left(\mu_{L}L+\alpha_{LI}IL\right)(1-\frac{I+L}{k}) \end{array} \end{array}$$ (2)

where *I* and *L* stand for the population density of the *I ^-^* and *L^-^* strain respectively, *t* and *r* are the time and spatial coordinates, *D* is the diffusion coefficient, *μ*_*i*_ is the Malthusian growth rate of species *i* ∈ [*I*, *L*], and *α*_*ij*_ (≥ 0) is the growth rate of species *i* assisted by its mutualistic partner *j* ∈ [*I*, *L*]. Note that, as in the case of the hypercycle model [30], an effective hyperbolic growth is confined to relatively low population densities by the carrying capacity *k*. The above set of equations generalised the two-member model by including, on the one hand, the spatial context (through the diffusion terms *D*∂^2^/∂*r*^2^) and, on the other, by considering both mutualistic (*α*_*ij*_ ≥ 0) and Malthusian ($0\leq \mu_{i}\leq \mu_{i_{C}}$) growth terms.

The above minimal model [Eq. set (2)] is able to provide some analytical estimations for the front speed of the bacterial mutualistic loop. On the one hand, if we consider the absence of either species in the set Eq (2), we recover the one-species Fisher RD model [46, 50] that leads to the well-known expression for the invasion speed:

$$\begin{array}{c} \begin{array}{c} c_{I_{F}}=2\sqrt{\mu_{I}D}\;\text{for}\;L=0,\\ c_{L_{F}}=2\sqrt{\mu_{L}D}\;\text{for}\;I=0 \end{array} \end{array}$$ (3)

Moreover, the Fisher speed establishes the asymptotic invasion speed for our two-species system in Eq. set (2) as *μ*_*i*_ >> *α*_*ij*_ (for *i* = *I*, *L* and *i* ≠ *j* = *I*, *L*). In the case of two purely competing species (*μ*_*i*_ > 0, and *α*_*ij*_ = 0) we should expect the front to propagate at the speed of the faster competitor because this species will be more efficient at conquering the available space at the edge of the population front. In contrast, for the case of two purely mutualistic species (i.e., a pure loop with *μ*_*i*_ = 0, and *α*_*ij*_ > 0), we derived the analytical solution for the invasion speed (see Methods):

$$\begin{array}{c} c=\sqrt{\frac{Dk\alpha_{IL}\alpha_{LI}}{2(\alpha_{IL}+\alpha_{LI})}} \end{array}$$ (4)

Our minimal model (2) thereby predicts two different invasion modes for our pair of mutualistic strains *I ^-^* and *L^-^*. Indeed, in the competition scenario, the invasion speed Eq (3) is governed by the growth rate at low population densities(which gives rise to a pulled front [47, 51, 52]). In contrast, the carrying capacity *k* appearing in Eq (4) is a hallmark of an invasion front governed by the growth dynamics at high population densities. This gives rise to a pushed front [47, 51, 52]: individuals at the edge of the front are pushed from the inside bulk where individuals reproduce at higher rates. Moreover, note that the invasion speed Eq (4) is the same for the two mutualists *I ^-^* and *L^-^*, consistent with their need for a mutualistic partner in order to grow and spread.

**Fig 3:**
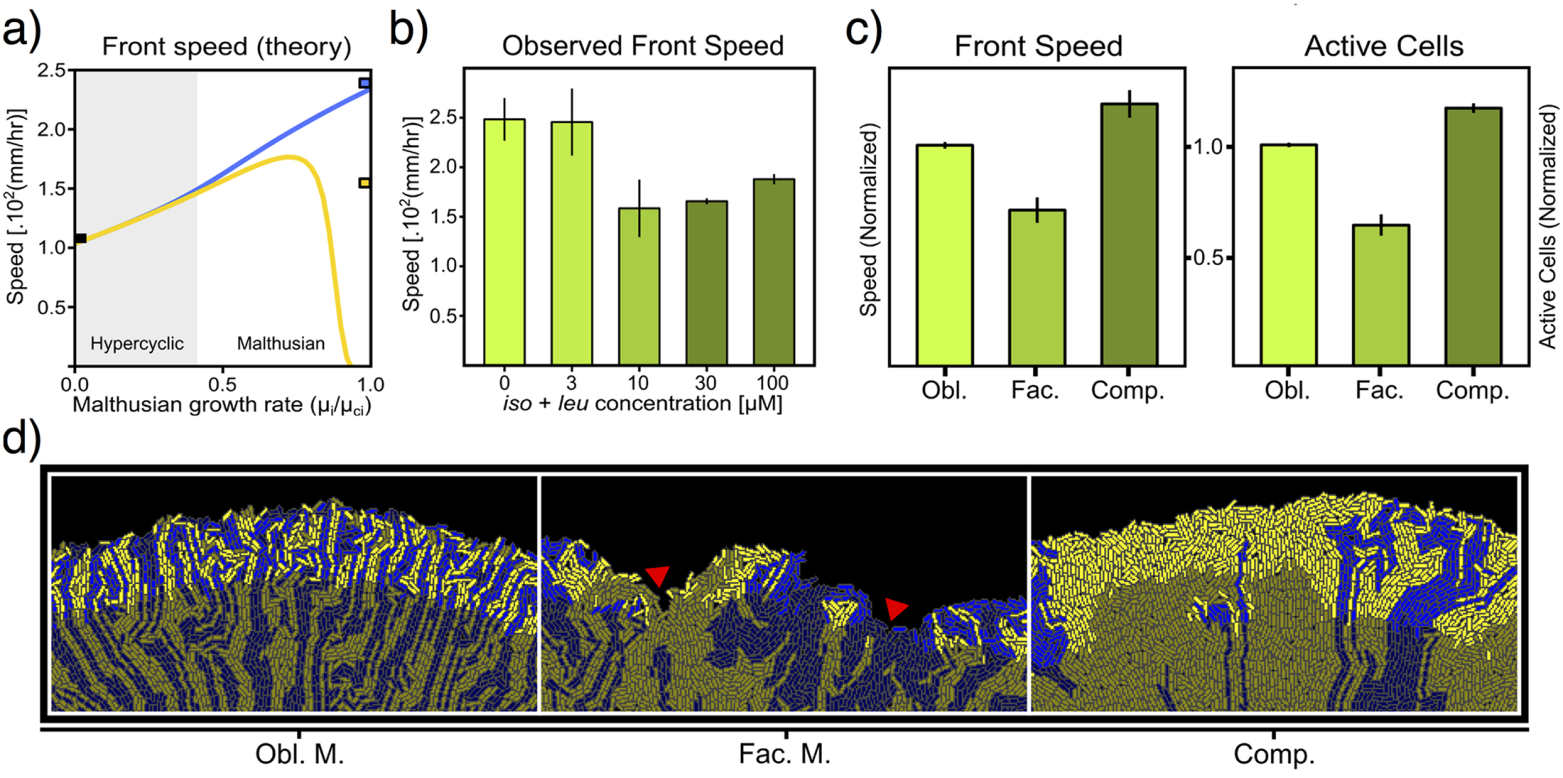
Improved environments can slow down the front of synthetic mutualists. *a)* Invasion speed of the mutualistic strains according to a minimal reaction-diffusion model. The gray area indicates the domain where the mutualistic interaction favours hyperbolic growth over Malthusian competition. The maximum Malthusian growth rates *μ*_*CI*_ = 9.13 × 10^−2^ and *μ*_*CL*_ = 2.18 × 10^−1^ hr^−1^ (for *I ^-^* and *L^-^*, respectively) correspond to monoculture growth rates observed in the competition scenario (see S1 Fig). *b)* Observed front speeds exhibit a slowing-down in facultative mutualism scenarios that is not captured by the RD model (average and standard deviation values over 5 replicates are shown). *c)* According to agent based simulations, the slow down in facultative mutualism scenarios is correlated with a decay in the fraction of active cells. *d)* Snapshots of simulated fronts (darker colours depict stagnant cells). The red arrow indicates a patch of *I ^-^* cells formed by local consumption of environmental amino acids. Once amino acids are locally depleted, a high number of cells in the patch become stagnant.

Fig 3a shows how the transition between the two invasion modes takes place, according to the RD model. In the absence of Malthusian replication (*μ*_*i*_ = 0), both strains spread at the same speed. As both *μ*_*I*_ and *μ*_*L*_ are increased towards their observed value (see S1 Fig) in the competition scenario, the front speed increases due to the corresponding enhancement in growth rates. However, once *μ*_*i*_ induces stronger effects on the front than *α*_*ij*_, competition becomes important and the coupled advance of the two strains is replaced by two differentiated front speeds. At this point, further increasing the Malthusian growth rates *μ*_*i*_ benefits the faster species (in this case, the *L^-^* strain), while the second one is slowed down in a relatively abrupt way (changes in the corresponding population density profiles are shown in S6 Fig). This eventually leads the *I ^-^* strain to be excluded from the front (which propagates at the Fisher’s speed $c_{L_{F}}$ as Malthusian growth rates approach the observed values in competition). It is worth noting that, according to the RD model, the minimal invasion speed of the population corresponds to that in the obligate mutualism scenario (i.e. any increase in Malthusian growth rates would lead to a faster population front, for at least one of the species).

Fig 3b reveals a slowdown in the invasion speed for facultative mutualists that the RD minimal model was unable to predict. We measured the front speed for cocultures spreading on agar surfaces (see Methods), observing particularly low values of the front speed at the transition between the obligate mutualism and the competition scenario. According to the RD model, even if one of the strains is slowed down because of competition, the edge of the front will keep travelling at the speed of the fastest strain (which should exceed the speed of the obligate mutualistic loop in order to overcome its partner species at the edge of the front). Thus, the decrease of the observed front speed as supplemented amino acids are increased indicates that other, more complex phenomena are driving the dynamics of the synthetic mutualistic feedback. In particular, the physical embodiment of bacterial cells (not taken into account by the RD model) may affect their access to the extracellular amino acids, thus influencing the invasion speed.

Local nutrient depletion leads to the range expansion slowdown of facultative mutualists. Simulations in Fig 3c and 3d capture a slowdown in the invasion speed similarly as observed in experimental conditions. As the snapshots in Fig 3d illustrate, nutrients and amino acids are mainly consumed by cells at the edge of the front, their depletion leaves a population of stagnant cells that effectively constitutes a fossil record of the invasion process [41]. In the obligate mutualism case, single-strain patches keep a characteristic width determined by the distance at which cells can sustain the cross-feeding mutualism (cells near the front can temporarily become stagnant when their location prevents an effective cross-feeding). This process shapes the spatial distribution of the population, leading to a relatively high fraction of active cells at the edge of the front (Fig 3c and 3d). However, in the case of facultative mutualism, the dynamics can be marked by episodes of opportunistic growth that exploits the available amino acids in the environment. During these periods, the dynamics are locally governed by genetic drift (single-strain sectors become wider). However, once the supplemented amino acids are locally depleted, a significant number of cells (remote to the boundary domains where cross-feeding is still effective) can become stagnant (arrow in Fig 3d). Fig 3c shows how the ratio of active cells is correlated with the invasion speed, suggesting that the dynamics in facultative mutualism scenarios can slow-down the invasion speed of the synthetic mutualists.

### Environmental deterioration can determine the survival of parasites during range expansions

**Fig 4:**
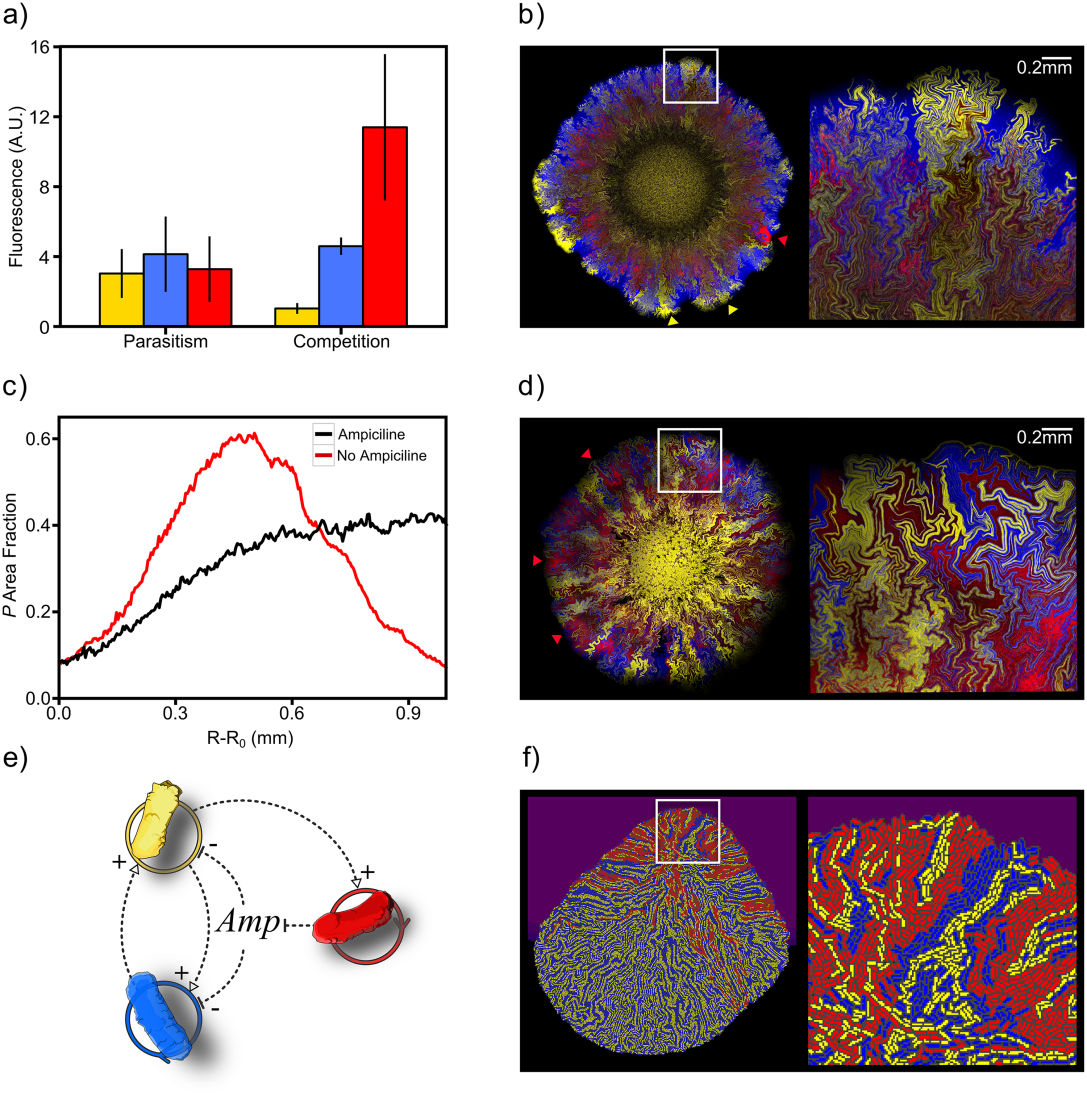
Environmental conditions determine the fate of parasites during range expansions. *a)* Obligate mutualism scenario (absence of supplemented amino acid) leads the strain *P* to act as a parasite in well-mixed conditions, while competition is observed at 10^−4^M supplementation of *iso* and *leu*. Average and standard deviation values over 9 replicates are shown. *b)* Spatial structure leads the mutualists to conquer the edge of the population front, defeating the parasite *P*. Yellow arrows indicate regions where the parasite has been excluded from the population front (red arrow indicates one of the few regions in which the parasite still surfs at the edge of the front). Note that the front curvature is enhanced at regions governed by the mutualists, a hallmark of an enhancement of the front speed at these regions. The grey rectangle indicates the magnified area on the right. *c)* Frequency of the *P* strain at the edge of the front for two different scenarios (0 and 100*μ*M extracellular ampicillin). *d)* The *P* strain offers cross-protection to the mutualists when threatened by antibiotics, leading to the survival of the *P* strain at the edge of the front. *e)* Scheme of the complex mutualistic interaction (which involves cross-feeding and cross-protection) between the three species in the presence of antibiotics. Each species lacks a different ability needed to survive in the system, but the ensemble may be able to survive if able to develop the corresponding division of labour. *f)* Three-species spatial structure in a simulated heterogeneous environment with non-isotropic antibiotic concentration at *t* = 0. While the *P* strain is conserved in the areas where cross-protection is essential for the mutualistic ensemble, *P* cells are excluded from the front in areas where the antibiotic concentration does not reach the growth inhibition threshold.

Several processes (such as mutations or the arrival of foreign, invader species) may give rise to new organisms exploiting cooperative feedbacks in a given ecosystem. The introduction of a new replicator organism that makes use of the limited resources in the medium will restrict the growth of the coupled system, specially if this new organism is a parasite (hereafter *P* cells) that takes advantage of the cross-feeding (Fig 4e).

In order to experimentally study the ecological implications of such parasites, we used the synthetic parasitic strain *P* (see Methods) that exploits one of the cross-feeding amino acids (namely, *iso*). The coculture of those three organisms in well-mixed conditions, for both the obligate mutualism and the competition scenarios, give as a result a restricted growth of *I ^-^* or *L^-^* strains (Fig 4a shows lower fluorescence values for both strains than those in Fig 2b). Moreover, for the competition scenario in Fig 4a, the *P* strain exhibits a relatively high Malthusian growth rate (see S1 Fig) that leads it to overcome the growth of the mutualistic pair.

To test whether spatial structure can limit the parasitic exploitation, we coculture combinations of the three strains (*I ^-^*, *L^-^* and *P*) on M63-agar plates. In the absence of supplemented amino acids, when *I ^-^* or *L^-^* cells are lacking, no growth was observed. This means that *P* cells can be considered a hypercycle parasite, because they are unable to close an effective cross-feeding loop (see Fig 2a) with either *I ^-^* or *L^-^* cells. When the three strains are present (Fig 4b), despite an initial success of the parasite at colonising available space (see Fig 4c, red line), the parasitic strain is progressively left behind as the range expansion takes place. This is because, in the spatial scenario, cell location determines a preferential access to the cross-feeding metabolites [44, 45]. Therefore, the presence of a *P* patch increases the distance between *I ^-^* and *L^-^* and leads to restricted growth. This gives a significant advantage to mutualistic *I ^-^* and *L^-^* neighbouring patches that engage in an efficient cross-feeding. Hence, spatial structure benefits the hypercycle species, eventually leading the hypercycle ensemble to overcome the parasite at the edge of the front (Fig 4b and S7 Fig).

The ecological role of a species in a given community can be strongly dependent on its environment and transitions can occur between mutualism and parasitism as external conditions change [2, 53–56]. In our three-member microbial consortium, composed by *I ^-^* *L^-^* and *P*, we studied whether environmental deterioration can make this community to develop a more complex mutualistic network. In order to do this, the three-member microbial consortium was seeded on m63-agar plates containing a lethal concentration of ampicillin, for which *P* cells are resistant. The *P* cells are able to degrade extracellular Ampicillin (by secreting beta-lactamase). Now, two different mutualistic motives are present in this scheme (Fig 4e): (amino acids) cross-feeding and (antibiotic) cross-protection. Remarkably, the hypercycle trio was able to solve the complex environmental problem and develop the range expansion process on the corresponding agar layers. Fig 4d shows the observed spatial structure displayed by this new mutualistic ensemble while invading the available space. In contrast to the previous parasitic case, the fraction of the *P* strain is approximately constant as the population front advances (see Fig 4c).

The definition of the three-member consortium as an agent-based model allows us to make some predictions on how the system would spread within heterogeneous environments and captures the main spatial dynamics features of the system (see Supp Info). Simulation in a heterogeneous environment, that presents an asymmetric spatial antibiotic distribution, allows us to see how the *P* strain remains present at the edge of the front in the top region of the colony, which is precisely where the population is exposed to higher doses of antibiotic. In contrast, in the lower region where the antibiotic dose is much lower, the *P* strain is excluded from the edge of the front (consistently with our previous results), (Fig 4f).

This is an interesting result particularly within the context of bioengineering soils [22, 23] by the rewiring of the ecological interactions within the biological soil crust (BSC). Here the vertical structure defines a heterogeneous set of conditions where different species and physicochemical spatial gradients are present. Both in the BSC and around the plant root system a complex microbiome exists. Soil engineering under a systems perspective is a promising domain to harness and restore different functionalities [57]. This approach could be complemented by designed microbiomes that exploit mutualistic ties following some of the basic findings reported here. Since different soil conditions might sustain different qualitative functional traits, the previous synthetic three-species ecosystem can inspire novel forms of improving soil communities and plant efficiency.

## Discussion

Most experimental and theoretical studies concerning the dynamics of microbial populations are grounded in competition. However, cooperation is a crucial component of ecological dynamics on all scales, and is much needed to truly understand the behaviour of a wide range of systems from populations growing on biofilms to the gut microbiome or even solid tumor ecosystems [58, 59] (in which multiple cancer strains can cooperate to succeed). Moreover, it has been suggested that synthetic cooperation can help to design ecological circuits capable of preventing endangered ecosystems from collapsing [22, 23].

Previous studies have analysed a family of models involving closed mutualistic loops. These systems are known as hypercycles, and because of their second-order kinetics, they are capable of hyperbolic growth, allowing the hypercycle to overcome the simple Malthusian replicators. Theoretical works show that hypercycles can prevent their own decay due to the presence of parasites by exploiting the constraints imposed by a spatially extended system. However, these models require some special properties concerning the nonlinear dynamics of hypercyclic sets, which are not feasible in realistic conditions. Instead, we have analysed persistence and response to parasites associated to this kind of systems by means of experimental setups where populations of engineered mutualists spread on a two-dimensional medium.

Our study reveals that, as predicted by theoretical models involving both linear (Malthusian) growth and hypercyclic cooperation, spatial dynamics (e.g. in the context of propagating fronts) can introduce critically important effects for the survival or extinction of hypercycle species. This is shown by both the microscopic impact of bacterial shapes (which can lead to characteristic boundary domains [38]) and by the local correlations required to sustain cooperation, which favour an enhancement of contact domains between the two cell populations. Hypercyclic growth has been characterised using diverse sets of metrics and the front speed mathematically derived from a diffusion model.

The experiments and models confirm the picture of spatial mutualists as dynamical systems where the mutualistic tie forces the formations of complex structures that guarantee the propagation of the cooperative consortium. We have also studied the tradeoffs associated with Malthusian growth and the conditions pervading the breakdown of hypercyclic cooperation thus showing the presence of two phases: one associated with competitive interactions and another phase associated with scarce resources promoting the mutualistic feedback. Interestingly, we have shown that, as the interactions transit from obligate mutualism to competition, population range expansions can be slowed-down despite the richer resource availability in the environment. In such richer environments, genetic drift especially influences the spatial structure (creating wide single-strain sectors) while the population exploits local resources [60]. This decreases the cross-feeding efficiency between mutualists, which can lead to slow down the front speed once resources are locally depleted.

The second set of experiments and models are related to the impact of parasitic strains on the stability of the hypercycle. We designed synthetic parasitic strains capable of exploiting a given amino acid while not completing the mutualistic cycle. Such parasite (which has a small component of Malthusian growth) has been shown to overcome and kill the hypercycle under liquid conditions but becomes a much less harmful component under spatial constraints. These results suggest that spatial constraints can favour mutualistic populations over parasitic mutants that are likely to arise [1, 44, 61] over evolutionary timescales. For cross-feeding mutualisms, parasitic mutants could avoid the cost of the mutualism by reducing (or cutting) the overproduction of mutualistic metabolites (here, aminoacids). Moreover, selfish mutants could follow alternative (perhaps additional) evolutionary routes leading to avoid the need for the mutualistic partner (e.g., by developing the ability to metabolize both essential amino acids). Despite the relatively short timescales involved in our experiments, we occasionally observed mutant sectors exhibiting a different spatial structure (S8 Fig) than the rest of the sectors in the colony (suggesting that the corresponding mutant strain modified its mutualistic interactions) [62]. It is worth noting that, as long as alternative ways to optimize growth rates are available, fitter mutants could also arise without changing their population interactions. For example, S8 Fig shows a case in which a mutant sector exhibited a cut in its fluorescent reporter, whose expression is metabolically costly.

Finally, we have shown that environmental deterioration (e.g., due to a toxic molecule) can reshape population interactions, leading this (otherwise parasitic) strain to become a member of a three-strain hypercycle. It was recently shown that resource availability can modulate the interactions between microbial cross-feeding mutualists [9, 43]. Our work is, as far as we know, the first experimental design of a synthetic ecological network showing how different contexts allow mutualism, competition or parasitism to succeed or even transition from one to the other in a spatially extended context. Further work should explore how these results translate into more realistic contexts, from the gut microbiome to soil ecosystems.

## Materials and methods

### Theoretical invasion speed of a 2-species hypercycle

Our theoretical RD model for the two-species hypercycle considers that the dynamics of the species *I*(**r**, *t*) and *L*(**r**, *t*) is governed by diffusion and population growth as:

$$\begin{array}{c} \begin{array}{c} \frac{\partial I}{\partial t}=D\nabla^{2}I+\left(\mu_{I}I+\alpha_{IL}IL\right)(1-\frac{I+L}{k}),\\ \frac{\partial L}{\partial t}=D\nabla^{2}L+\left(\mu_{L}L+\alpha_{LI}IL\right)(1-\frac{I+L}{k}). \end{array} \end{array}$$ (5)

For simplicity, we have neglected the death rates in Eq. set (1), considering that the logistic term sufficiently captures growth inhibition effects (as it is a standard approach when studying biological range expansions [47]). Moreover, we are interested in the asymptotic front speed (*r* → ∞ and *t* → ∞) for the case of short-range, isotropic migration. Thus, the Laplacian in polar coordinates simplifies into:

$$\begin{array}{c} \begin{array}{c} \nabla^{2}U=\frac{1}{r}\frac{\partial }{\partial r}(r\frac{\partial U}{\partial r})+\frac{1}{r^{2}}\frac{\partial U}{\partial \theta^{2}}≃\frac{\partial^{2}I}{\partial r^{2}} \end{array} \end{array}$$ (6)

which leads us to Eq (2), i.e.:

$$\begin{array}{c} \begin{array}{c} \frac{\partial I}{\partial t}=D\frac{\partial^{2}I}{\partial r^{\;2}}+\left(\mu_{I}I+\alpha_{IL}IL\right)(1-\frac{I+L}{k}),\\ \frac{\partial L}{\partial t}=D\frac{\partial^{2}L}{\partial r^{\;2}}+\left(\mu_{L}L+\alpha_{LI}IL\right)(1-\frac{I+L}{k}). \end{array} \end{array}$$ (7)

For convenience, we rewrite this set of equations in terms of dimensionless variables I* = *I*/*k*, L* = *L*/*k*, t* = *α*_*IL*_*kt* and r* = (*α*_*IL*_*k*/*D*)^1/2^*r*, and dimensionless parameters α* = *α*_*LI*_*k*/*α*_*IL*_. Thus, the new set reads:

$$\begin{array}{c} \frac{dI^{*}}{dt^{*}}=\frac{\partial^{2}I^{*}}{\partial r^{*2}}+I^{*}L^{*}\left(1-I^{*}-L^{*}\right) \end{array}$$ (8)

$$\begin{array}{c} \frac{dL^{*}}{dt^{*}}=\frac{\partial^{2}I^{*}}{\partial r^{*2}}+\alpha^{*}I^{*}L^{*}\left(1-I^{*}-L*\right), \end{array}$$ (9)

Let us assume that there exist travelling wave-shaped solutions of the previous equations of the form:

$$\begin{array}{c} I^{*}\left(r^{*},t^{*}\right)=U_{I}\left(z\right)=\xi_{I}\frac{1}{{\left(1+ae^{bz}\right)}^{s}}, \end{array}$$ (10)

$$\begin{array}{c} L^{*}\left(r^{*},t^{*}\right)=U_{L}\left(z\right)=\xi_{L}\frac{1}{{\left(1+ae^{bz}\right)}^{s}}, \end{array}$$ (11)

with *s* > 0, *b* > 0, *a* > 0, and *z* = *r* − *ct* (where *c* is the speed of the travelling wave, i.e. the front speed of the hypercyclic population). Using

$$\begin{array}{c} \frac{dU_{i}}{dx}=\frac{dU_{i}}{dz}=U_{i}^{\prime } \end{array}$$

$$\begin{array}{c} \frac{dU_{i}}{dt}=-c\frac{dU_{i}}{dz}=cU_{i}^{\prime } \end{array}$$

with *i* ∈ [*I*, *L*], the set Eq (9) can be rewritten as:

$$\begin{array}{c} U_{I}^{\prime \prime }+cU_{I}^{\prime }+U_{I}U_{L}\left(1-U_{I}-U_{L}\right)=0 \end{array}$$ (12)

$$\begin{array}{c} U_{L}^{\prime \prime }+cU_{L}^{\prime }+\alpha^{*}U_{I}U_{L}\left(1-U_{I}-U_{L}\right)=0, \end{array}$$ (13)

Developing the derivatives $U_{I}^{\prime \prime }$ and $U_{I}^{\prime }$, Eq (12) reads:

$$\begin{array}{c} \begin{array}{c} \varepsilon_{I}[s\left(s+1\right)\eta^{-s-2}a^{2}b^{2}e^{2bz}-s\eta^{-s-1}ab^{2}e^{bz}\\ -sc\eta^{-s-1}abe^{bz}\\ +\varepsilon_{L}\eta^{-2s}-\varepsilon_{I}\varepsilon_{L}\eta^{-3s}-\varepsilon_{L}^{2}\eta^{-3s}]=0, \end{array} \end{array}$$ (14)

where *η* = (1 + *ae*^*bz*^). Neglecting the trivial solution (*ε*_*I*_ = 0) for Eq (14), and reorganising terms according to powers of *e*^*bz*^, we obtain the characteristic equation for the front speed *c*:

$$\begin{array}{c} \begin{array}{c} e^{2bz}\left[s\left(s+1\right)a^{2}b^{2}\right]+e^{bz}\left[-sa\eta \left(b^{2}+bc\right)\right]\\ +\varepsilon_{L}\eta^{-s+2}+\varepsilon_{I}\varepsilon_{L}\eta^{-2s+2}+\varepsilon_{L}^{2}\eta^{-2s+2}=0 \end{array} \end{array}$$ (15)

Solutions for the travelling wave have to be valid ∀*z*, and thus each line in Eq (15) gives an independent expression that must necessarily vanish. Analysing the terms in the last line in Eq (15) leads to the necessary condition *s* < 2. This leads to *s* = 1 because we only consider solutions with *s* > 0. Then, considering *s* = 1, we develop the conditions given by the different powers of *e*^*bz*^ in Eq (15), which leads to:

$$\begin{array}{c} \varepsilon_{I}=1-\varepsilon_{L}, \end{array}$$ (16)

$$\begin{array}{c} c=\frac{\varepsilon_{L}-b^{2}}{b}, \end{array}$$ (17)

and

b=c. (18)

Combining Eqs (16)–(18) leads to:

$$\begin{array}{c} c=\sqrt{\varepsilon_{L}/2}=\sqrt{(1-\varepsilon_{I})/2}. \end{array}$$ (19)

With an analogous procedure to the one performed above for Eq (12), analysis of Eq (13) leads to:

$$\begin{array}{c} c=\sqrt{\alpha^{*}\varepsilon_{I}/2}=\sqrt{\alpha^{*}\left(1-\varepsilon_{L}\right)/2}. \end{array}$$ (20)

Combining Eqs (19) and (20) we obtain the expressions for the species abundances in the travelling front:

$$\begin{array}{c} \begin{array}{c} \varepsilon_{I}=1/\left(1+\alpha^{*}\right),\\ \varepsilon_{L}=\alpha^{*}/\left(1+\alpha^{*}\right) \end{array} \end{array}$$ (21)

Replacing terms from Eqs (21) into (20), we obtain the analytical solution for the front speed in dimensionless variables:

$$\begin{array}{c} c=\sqrt{\frac{\alpha^{*}}{2(1+\alpha^{*})}}. \end{array}$$ (22)

Finally, recovering dimension variables, the speed of the front reads:

$$\begin{array}{c} v=c\sqrt{Dk\alpha_{IL}}=\sqrt{\frac{Dk\alpha_{IL}\alpha_{LI}}{2(\alpha_{IL}+\alpha_{LI})}} \end{array}$$ (23)

### The agent based model

Our approach to the study of hypercycles reveals the importance of considering cells as embodied entities, both as interacting elements on a microscopic scale and as spatially extended populations. Moreover, cells need to incorporate the molecular circuits associated to the specific regulatory mechanisms along with chemical reactions, spatial diffusion and molecular signalling. To this goal, we used the specification language *gro* [63] as the platform for individual-based simulation of growing populations.

Our model integrates the main physical features of bacterial shape and growth [63], as well as the cross-feeding and cross-protection interaction between *I ^-^* *L^-^* and *P* strains. We used a very simple approach that considers a few step (Heavyside) functions to emulate cell behaviour. A list of the considered cell behaviour features follows:

1. Sensing: at each time step, each cell senses the extracellular concentration of three kinds of molecules: amino acids (*I ^-^* cells sense *iso*, while *L^-^* and *P* cells sense *leu*), food (this category embraces any other nutrients that cells may need to grow), and antibiotic (i.e., ampicillin).
2. Growth: cells grow (increase their cell volume) and divide at the realistic speed proposed in Ref. [63], provided that:
  - food concentration exceeds a given threshold value *g*_*f*_.
  - the corresponding amino acid (according to cell strain) exceeds a given threshold value *g*_*am*_.
  - antibiotic concentration is below a given inhibitory threshold *g*_*at*_.
  Accordingly, cell growth is arrested whenever any of the above conditions are violated.
3. Cells absorb extracellular food and release amino acid (or *β*-lactamase) at constant rates, provided that extracellular food exceeds *g*_*f*_. Specifically, *I ^-^* cells release *leu*, *L^-^* cells release *iso*, and *P* cells release the betalactamase enzime (that degrades the antibiotic) to the extracellular medium. Provided that growth conditions are satisfied, cells will also absorb the amino acid they need.

**Fig 5:**
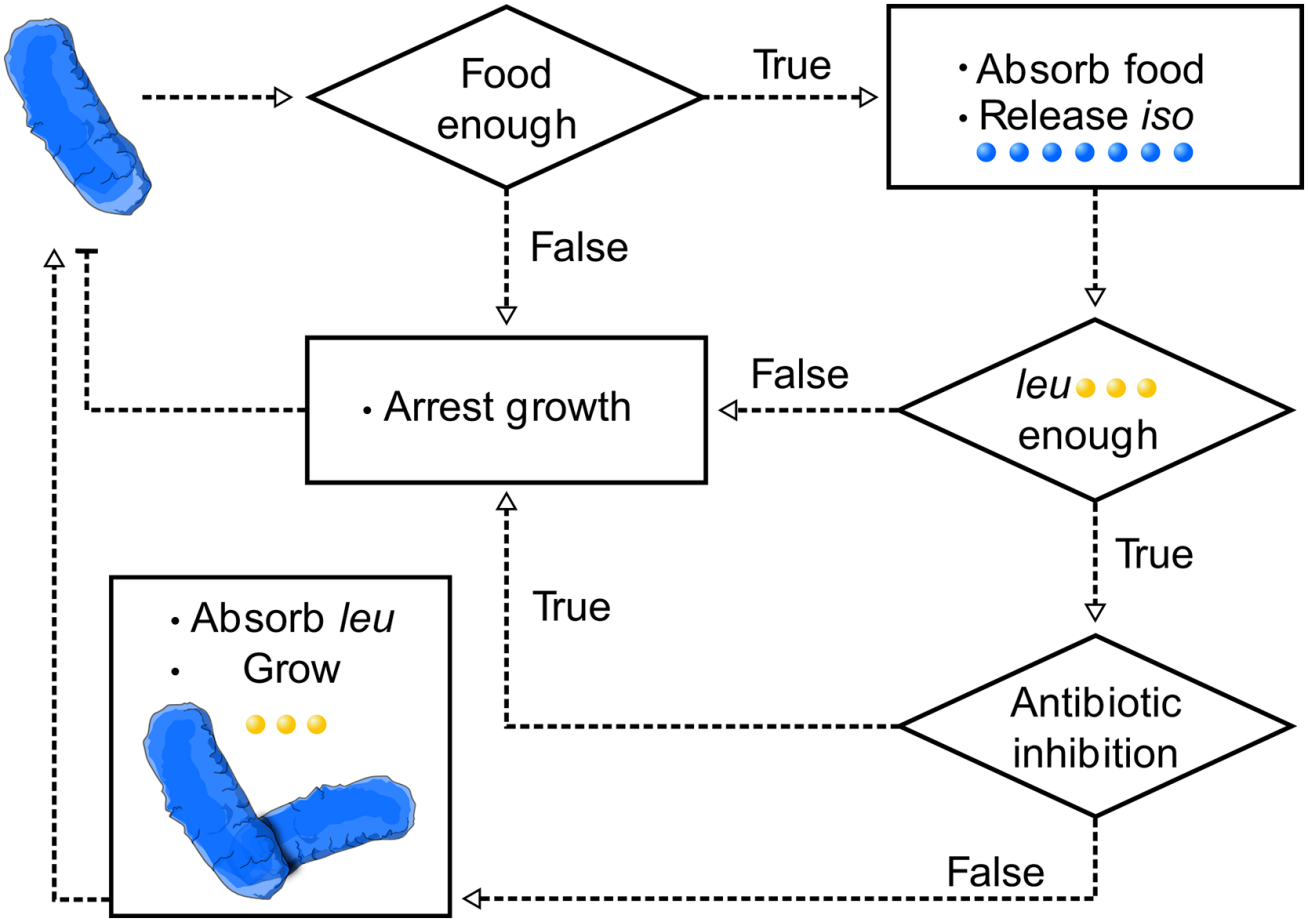
Cell dynamics in the agent-based model is governed by binary decisions (Heaviside behaviour) that depend on extracellular concentration thresholds of nutrients, amino acids and antibiotics. The scheme shows the logical steps that determine cell behavior according to our model.

The corresponding logical loop experienced by a given *L^-^* cell at each time step is illustrated in Fig 5. *I ^-^* and *P* cell dynamics follow analogous logical schemes.

Furthermore, in order to consider a fitter parasitic strain that evades the cost of the mutualism in antibiotic-free scenarios, we consider the growth rate of *P* cells to be higher (by a 10% difference) than that of *I ^-^* and *L^-^* cells. As shown above, the hypercycle was able to escape the parasite despite such faster growth rate.

Admittedly, actual cell dynamics is far more complex than this Heavyside representation. However, our goal for the agent-based model was to use a minimal set of assumptions, in order to provide an easy understanding of the key features governing the system dynamics. Remarkably, the Heavyside-based cell behaviour is enough to capture the essential dynamics, as discussed in the Results section. The source code and additional details on specific values for metabolic rates and concentration threshold values can be found in the Supp. Info.

### Bacterial strains

Both the *I ^-^* and the *L^-^* strains are from E. coli strain DH1 (National BioResource Project, National Institute of Genetics, Shizuoka, Japan) and were genetically modified to cross-feed as described in [6]. The *I ^-^* (*L^-^*) strain carries the *dsred.T3 (gfpuv5)* gene that provides the corresponding fluorescence labelling.

Cloning for the *P* strain was carried out using the Biobrick assembly method and the parts: B0014, J23100, B0032 and E0020, from the Spring 2010 iGEM distribution assembled into a low copy number plasmid pSB4A5. A complete description of the construction protocols can be found at [64, 65].

### Culture conditions

All regular cultures and amplifications were done at 37°C in well-mixed media Lysogeny Broth (LB). Bacterial strains were cryopreserved in LB-glycerol 20% (v/v) at -80°C. Along experiments, cells were grown at 37°C in well-mixed Modified-M63 (mM63) media (pH 7.0, 62 mM *K*_2_*HPO*_4_, 39 mM *KH*_2_*PO*_4_, 15 mM ammonium sulfate, 1.8 *μ*M *FeSO*_4_ − 7*H*_2_*O*, 15 *μ*M thiamine hydrochloride, 0.2 mM *MgSO*_4_ − 7*H*_2_*O* and 22 mM glucose [66]).

For individual cloning selection, *I*^-^ and *L*^-^ cells from frozen stocks were grown overnight 16h in LB at 37°C, diluted and plated on Petri dishes with LB agar (1.2% agar) and the appropriate selective antibiotic (chloramphenicol 30 *μ*g/ml, kanamycin 20 *μ*g/ml, and 25 *μ*g/ml for the for the *I ^-^*, the *L^-^*, and the *P* strain, respectively).

Before each experiment, colonies of each strain were selected and grown separately in LB supplemented with both 10^−^4M of auxotrophic amino acid and the corresponding selective antibiotic. After 16h overnight culture at 37°C, we performed a 100-fold dilution (500-fold in the case of *P* strain cultures) into fresh LB (supplemented with auxotrophic amino acid and selective antibiotic), and let cultures grow to OD660∼0.4.

### Fluorescence assays

Fresh cultures at OD660∼0.4 were washed twice using mM63 medium. In order to set the initial cell density for experiments, optical density was adjusted to *OD*660*nm* = 0.1 per strain (which means that cocultures involving 2 or 3 strains exhibited *OD*660*nm* = 0.2 or *OD*660*nm* = 0.3, respectively) after culture washing. Well-mixed culture experiments were performed in flat bottom 96-well microplates (Sarstedt AG & Co. Germany). Growth was monitored over time, by quantification of fluorescence identifying each strain (mRFP, GFP, and CFP for *I*^-^, *L*^-^, and *P* cells, respectively). M63 without cells was included in the incubation as a background control for both fluorescence and absorbance. Fluorescence time courses for well-mixed cultures were performed on a Synergy MX-microplate reader (BioTek Instruments, USA), using the reading settings for RFP (ex: 560±9 nm, em: 588±9 nm), GFP (ex: 478±9 nm, em: 517±9 nm) and CFP (ex: 450±9 nm, em: 476±9 nm) at gain 90, as well as optical density (OD at 660 nm). Incubation was performed at 37°C with continuous orbital shaking (medium speed).

### Range expansions on agar surfaces

Fresh cultures at OD660∼0.4 were washed twice using mM63 medium, and then resuspended in mM63 medium while adjusting the *OD*660*nm* = 0.15 per bacterial strain, in order to adjust the initial cell density for experiments. For range expansions in environments including ampiciline, we used an initial *OD*660*nm* = 0.3 per bacterial strain. 0.4 *μ*L of the corresponding cultures where then inoculated in mM63 1.2% agar plates (supplemented with amino acid and antibiotic as required by the experimental scenario). Colonies were incubated for 4 days (7 days for the case of front speed measurements) at 37°C and humidity 90%.

Colonies were observed using a Leica TCS SP5 AOBS (inverted) confocal microscope.

## Supporting information

S1 Fig Malthusian and hypercycle growth rates for the synthetic strains. *a)* Time series for the fluorescence of the *I ^-^* strain, when cultured in M63 medium supplemented with 100 *μ*M of both *iso* and *leu*. Coloured dots stand for the average values across 9 replicates (three technical replicates from each of three biological replicates), shaded area indicates standard deviation. The Malthusian growth rate *μ*_*I*_ was obtained by linear regression (black solid line) to the data during the exponential growth regime (region delimited by the vertical dashed lines), as described in S1 Text. *b)* Malthusian growth rate for the *L^-^* strain (growth conditions as in a)). *c)* Malthusian growth rate for the *P* strain (growth conditions as in a)). Hyperbolic growth rates *α*_*IL*_ and *α*_*LI*_ were obtained from the observed growth at low population densities (region between dashed lines), as described in S1 Text. The time series correspond to the growth of both *I ^-^* and *L^-^* strains in coculture, in M63 medium with no supplemented amino acids.
(TIFF)

S2 Fig Cell concentration scales linearly to fluorescence for the three species. *a)* Cell concentration in liquid cultures of the *I ^-^* strain according to their fluorescence. The value of *a* indicates the slope (in ml^−1^) obtained by linear regression of the data points. *b)* In agreement with cell concentration, optical density also scales linearly to fluorescence for the *I ^-^* strain. *c)* and *d)* show the same analysis as in *a)* and, but for the *L^-^* (while *e)* and *f)* correspond to analogous results for the *P* strain).
(TIFF)

S3 Fig Spatial structure close to the edge of the population front after four days of incubation. Different concentrations of supplemented *iso* and *leu* lead to different spatial dynamics at the edge of the front (e.g., [*iso*] = 0 and *leu* = 10^−4^M leads the *L^-^* strain to govern the front). White rectangles indicate the obligate mutualism, facultative mutualism and competition scenarios.
(TIFF)

S4 Fig Agent-based simulations capture the spatial dynamics of hypercycle range expansions. *a)* Agent-based simulations show analogous scenarios to those observed in Fig 3a. Values on the vertical and horizontal axis indicate the parameter values for the initial extracellular concentration of amino acids (*I*0 and *L*0, respectively, see S1 Table). b) Patch width in simulated range expansions, for a different initial extracellular concentration of amino acids (initial nutrient concentration *F*0 = 90). c) A biological replicate for each of the cases presented in Fig 3c in the Main Text.
(TIFF)

S5 Fig Cell shape influences mesoscopic boundary domains. *a)* Fractal dimension for the boundaries between *I ^-^* and *L^-^* patches in the obligate mutualism scenario. Bars indicate average values, while vertical lines indicate standard deviation from three different simulations. *b)* A snapshot showing the patches of the *I ^-^* strain (in white), when de division size parameter is set to 2.0, for a colony with approximately 1.6 × 10^4^ individuals. *c)* A snapshot showing the patches of the *I ^-^* strain (in white), when de division size parameter is set to 3.5, for a colony with approximately 1.6 × 10^4^ individuals.
(TIFF)

S6 Fig Front shape from reaction-diffusion model for hypercycles. Population density profiles during range expansion of hypercycle strains for different Malthusian growth rates (which models the effect of supplemented amino acids in the medium). The top panel shows the obligate (*μ*_*i*_ = 0) hypercycle case: the coupled populations propagate as two travelling waves that approximately share the location of their fronts’ edge. In the medium panel (*μ*_*i*_ = *μ*_*Ci*_/2), the two species display interactions at the critical intersection that separate mutualism from competition: both strains travel at similar speeds, but the front edge of *I ^-^* remains slightly behind one of *L^-^* due to its smaller growth rate in the presence of amino acids. In the lower panel (*μ*_*i*_ = *μ*_*Ci*_), the faster replicator *L^-^* wins the competition by conquering the available space long before *I ^-^*, which is progressively let behind until it is excluded from the population range expansion process.
(TIFF)

S7 Fig Fraction of *P* strain in range expansions. *a)* In silico, fraction of territory colonized by *P* cells in three-species population range expansions (curves show average values over 5 simulations). Three different scenarios are shown: no ampicillin (*Ampi*0 = 0.0, see S1 Table), moderate ampicillin concentration (*Ampi*0 = 2.0), and high ampicillin concentration (*Ampi*0 = 4.0). *b)* Biological replicate for the two scenarios in Fig 4c.
(TIFF)

S8 Fig Mutant sectors occasionally arised during experiments. Such mutant sectors were infrequent (less than one mutant sector per colony on average) and were not taken into account for the analysis in the Main text. *a)* The arrow indicates a mutant sector that reached a significantly wider length than the average length for a *L^-^* sector in the colony (obligate mutualism scenario). *b)* Mutant sector from the *P* strain exhibiting reduced fluorescent protein expression.
(TIFF)

S1 Table Relevant parameters in the agent-based model. The table shows the main parameters of the agent-based model, as well as the main processes they affect. Unless stated otherwise in the text, the parameter values used in simulations correspond to those in the source code (S2 Text).
(PDF)

S1 Text Growth rates in well-mixed conditions. Approximations for low-density population dynamics used to infer Malthusian and hyperbolic growth rates from experimental data in well-mixed conditions.
(PDF)

S2 Text Agent based simulations source code. Source code used to run our simulations in the gro package [63].
(PDF)

S3 Text Front speed for one-species hypercycles. Derivation of the theoretical front speed for one-species hypercycles.
(PDF)

## References

[1] Maynard-Smith J. and Szathmàry E. (1995) *The major transitions in evolution*. Oxford U. Press, Oxford.

[2] Bronstein J. L. (Ed.). (2015). Mutualism. Oxford University Press, USA.

[3] Darwin C. (1892). The formation of vegetable mould, through the action of worms, with observations on their habits. J. Murray, London UK.

[4] Wilkinson D. M. (2006). Fundamental processes in ecology: an earth systems approach. Oxford University Press

[5] Momeni B., Chen C. C., Hillesland K. L., Waite A., and Shou W. 2011 Using artificial systems to explore the ecology and evolution of symbioses. Cell. Mol. Life Sci. 68, 1353–1368. 10.1007/s00018-011-0649-y 21424911 PMC11114700

[6] Hosoda K., Suzuki S., Yamauchi Y. et al 2011 Cooperative adaptation to establishment of a synthetic bacterial mutualism. PLoS One 6, e17105 10.1371/journal.pone.0017105 21359225 PMC3040204

[7] Celiker H. and Gore J. 2013 Cellular cooperation: insights from microbes. Trends Cell Biol 23, 9–15. 10.1016/j.tcb.2012.08.010 22999189

[8] Shou W., Ram S. and Vilar J. M. 2007 Synthetic cooperation in engineered yeast populations. Proc. Natl. Acad. Sci. USA 104, 1877–1882. 10.1073/pnas.0610575104 17267602 PMC1794266

[9] Hoek TA, Axelrod K, Biancalani T, Yurtsev EA, Liu J, Gore J. (2016) Resource Availability Modulates theCooperative and Competitive Nature of a Microbial Cross-Feeding Mutualism. PLoS Biol 14(8): e1002540 10.1371/journal.pbio.1002540 27557335 PMC4996419

[10] Wintermute E. H. and Silver P. A. 2010 Emergent cooperation in microbial metabolism. Mol. Sys. Biol. 6(1), 407. 10.1038/msb.2010.66 PMC2964121 20823845

[11] Agapakis C. M., Niederholtmeyer H., Noche R. R., Lieberman T. D., et al 2011 Towards a synthetic chloroplast. PLoS One 6, e18877 10.1371/journal.pone.0018877 21533097 PMC3080389

[12] Kiers ET, Duhamet M, Beesetty Y et al 2011 Reciprocal rewards stabilize cooperation in the mycrorrizal symbiosis. Science 333, 880–882. 10.1126/science.1208473 21836016

[13] Alvarez M., Reynaert N., Chávez M. N. et al 2015 Generation of Viable Plant-Vertebrate Chimeras. PloS one 10, e0130295 10.1371/journal.pone.0130295 26126202 PMC4488345

[14] Guan S. H., Gris C., Cruveiller S., Pouzet C et al 2013 Experimental evolution of nodule intracellular infection in legume symbionts. The ISME journal 7, 1367–1377. 10.1038/ismej.2013.24 23426010 PMC3695290

[15] Hom E. F., and Murray A. W. 2014 Niche engineering demonstrates a latent capacity for fungal-algal mutualism. Science 345, 94–98. 10.1126/science.1253320 24994654 PMC4409001

[16] Solé R. (2016). Synthetic transitions: towards a new synthesis. Phil. Trans. R. Soc. B, 371, 20150438. 10.1098/rstb.2015.0438 PMC4958932 27431516

[17] Weber B., Büdel B. and Belnap J. (2014). Biological soil crusts: an organizing principle in drylands. Springer-Verlag, Berlin.

[18] Maestre F. T., Eldridge D. J., Soliveres S., Kéfi S.et al (2016). Structure and Functioning of Dryland Ecosystems in a Changing World. Annual Review of Ecology, Evolution, and Systematics, 47(1). 10.1146/annurev-ecolsys-121415-032311 28239303 PMC5321561

[19] Van Der Heijden M. G., Bardgett R. D. and Van Straalen N. M. (2008). The unseen majority: soil microbes as drivers of plant diversity and productivity in terrestrial ecosystems. Ecology letters, 11(3), 296–310. 10.1111/j.1461-0248.2007.01139.x 18047587

[20] Folke C., Carpenter S., Walker B., Scheffer M., Elmqvist T., Gunderson L. and Holling C. S. (2004). Regime shifts, resilience, and biodiversity in ecosystem management. Ann. Rev. Ecol. Evol. Syst. 35, 557–581. 10.1146/annurev.ecolsys.35.021103.105711

[21] Solé R. 2007 Scaling laws in the drier. Nature 449, 151–153. 10.1038/449151a 17851503

[22] Solé R. 2015 Bioengineering the biosphere?. Ecological Complexity, 22, 40–49. 10.1016/j.ecocom.2015.01.005

[23] Solé S., Montanez R., and Duran Nebreda S. 2015 Synthetic circuit designs for earth terraformation. Biology Direct. 10: 37 10.1186/s13062-015-0064-7 26187273 PMC4506446

[24] Eigen M. and Schuster P. (1978). The hypercycle. Naturwissenschaften, 65(1), 7–41. 10.1007/BF00420631

[25] Kauffman S.A. The origins of order. Oxford Univ. Press, Oxford, 1993.

[26] Szathmáry E. 2006 The origin of replicators and reproducers. Phil. Trans. Royal Soc. London B 361, 1761–1776. 10.1098/rstb.2006.1912 PMC1664675 17008217

[27] Higgs P. G., and Lehman N. (2015). The RNA World: molecular cooperation at the origins of life. Nat. Rev. Genet. 16, 7–17. 10.1038/nrg3841 25385129

[28] Schuster P. (2016). Some mechanistic requirements for major transitions. Phil. Trans. Royal Soc. London B 371, 20150439 10.1098/rstb.2015.0439 PMC4958933 27431517

[29] Sardanyes J., and Solé R. (2006). Bifurcations and phase transitions in spatially extended two-member hypercycles. J. Theor. Biol. 243(4), 468–482. 10.1016/j.jtbi.2006.07.014 16942781

[30] King G.A.M. 1981 Growth of a hypercycle and comparison with conventional autocatalysis. Biosystems 13(4), 225–234. 10.1016/0303-2647(81)90001-0 7248484

[31] Smith J. M. 1979 Hypercycles and the origin of life. Nature, 280, 445–446. 10.1038/280445a0 460422

[32] Boerlijst M. C. and Hogeweg P. 1991 Spiral wave structure in pre-biotic evolution: hypercycles stable against parasites. Physica D 48(1), 17–28. 10.1016/0167-2789(91)90049-F

[33] Cronhjort M. B. and Blomberg C. 1994 Hypercycles versus parasites in a two dimensional partial differential equations model. J. Theor. Biol 169(1), 31–49. 10.1006/jtbi.1994.1128

[34] Attolini C. S. O. and Stadler P. F. 2006 Evolving towards the hypercycle: A spatial model of molecular evolution. Physica D 217(2), 134–141. 10.1016/j.physd.2006.03.015

[35] Sardanyés J. and Solé R. (2007). Spatio-temporal dynamics in simple asymmetric hypercycles under weak parasitic coupling. Physica D 231, 116–129. 10.1016/j.physd.2007.04.009

[36] May, Robert M. Hypercycles spring to life. Nature, 1991, vol. 353, no 6345, p. 607–608. 10.1038/353607a0

[37] Szathmáry E. 2013 On the propagation of a conceptual error concerning hypercycles and cooperation. J. Systems Chem. 4(1), 1 10.1186/1759-2208-4-1

[38] Rudge Timothy J., et al Cell polarity-driven instability generates self-organized, fractal patterning of cell layers. ACS synthetic biology, 2012, vol. 1, no 12, p. 365–374. 23688051 10.1021/sb400030p

[39] Smith WPJ, Davit Y, Osborne JM, Kim W, Foster K, and Pitt-Francis JM. 2017 Cell morphology drives spatial patterning in microbial communities. PNAS 114 (3), E280–E286. 10.1073/pnas.1613007114 28039436 PMC5255625

[40] Momeni B, Waite AJ, Shou W. 2013 Strong inter-population cooperation leads to partner intermixing in microbial communities. eLife 2013;2:e00230 10.7554/eLife.00230 23359860 PMC3552619

[41] Hallatscheck Oskar, et al Genetic drift at expanding frontiers promotes gene segregation. Procs. Natl. Acad. Sci. USA 2007, vol. 104, no 50, p. 19926–19930. 10.1073/pnas.0710150104 PMC2148399 18056799

[42] Hallatscheck Oskar; Nelson David, R. Life at the front of an expanding population. Evolution, 2010, vol. 64, no 1, p. 193–206. 10.1111/j.1558-5646.2009.00809.x 19682067

[43] Müller Melanie JI, et al Genetic drift opposes mutualism during spatial population expansion. Proceedings of the National Academy of Sciences, 2014, vol. 111, no 3, p. 1037–1042. 10.1073/pnas.1313285111 PMC3903240 24395776

[44] Momeni B, Waite AJ, Shou W. 2013 Spatial self-organization favors heterotypic cooperation over cheating. eLife 2013;2:e00960 10.7554/eLife.00960 24220506 PMC3823188

[45] Pande S, Kaftan F, Lang S, Svatos A, Germerodt S and Kost C. 2016 Privatization of cooperative benefits stabilizes mutualistic cross-feeding interactions in spatially structured environments. ISME 10, 1413–1423. 10.1038/ismej.2015.212 PMC5029186 26623546

[46] Fisher Ronald Aylmer. The wave of advance of advantageous genes. Annals of eugenics, 1937, vol. 7, no 4, p. 355–369. 10.1111/j.1469-1809.1937.tb02153.x

[47] Murray James D. Mathematical Biology I: An Introduction. Springer, Berlin, 2004.

[48] Korolev KS. (2013). The fate of cooperation during range expansions. PLOS Comp Biol 9, e1002994 10.1371/journal.pcbi.1002994 PMC3610630 23555227

[49] Isern N, Zilhao J, Fort J, Ammerman AJ. 2017 Modeling the role of voyaging in the coastal spread of the Early Neolithic in the West Mediterranean. PNAS 114 (5), 897–902. 10.1073/pnas.1613413114 28096413 PMC5293084

[50] Kolmogorov AN, Petrovsky N and Piscounov NS 1937 A study of the equation of diffusion with increase in the quantity of matter and its application to a biological problem Moscow Univ. Bull. Math. 1 1

[51] Gandhi SR, Yurtsev EA, Korolev KS, Gore J (2016) Range expansions transition from pulled to pushed waves as growth becomes more cooperative in an experimental microbial population. Proceedings of the National Academy of Sciences, 2016, vol 113, no 25, p. 6922–6927. 10.1073/pnas.1521056113 PMC4922184 27185918

[52] van Saarloos W (2003) Front propagation into unstable states. Phys Rep 386, 29–222. 10.1016/j.physrep.2003.08.001

[53] Bronstein J. L. (1994). Conditional outcomes in mutualistic interactions. Trends Ecol. Evol. 9, 214–217. 10.1016/0169-5347(94)90246-1 21236825

[54] Hernandez M. J. (1998). Dynamics of transitions between population interactions: a nonlinear interaction alpha-function defined. Proceedings of the Royal Society of London B 265, 1433–1440. 10.1098/rspb.1998.0454

[55] Herre E. A., Knowlton N., Mueller U. G. and Rehner S. A. (1999). The evolution of mutualisms: exploring the paths between conflict and cooperation. Trends Ecol. Evol. 14, 49–53. 10.1016/S0169-5347(98)01529-8 10234251

[56] Neuhauser C. and Fargione J. E. (2004). A mutualism-parasitism continuum model and its application to plant-mycorrhizae interactions. Ecological modelling 177, 337–352. 10.1016/j.ecolmodel.2004.02.010

[57] DeJong J. T., Soga K., Banwart S. A., Whalley W. R. et al (2010). Soil engineering in vivo: harnessing natural biogeochemical systems for sustainable, multi-functional engineering solutions. J. Roy. Soc. Interface 8, 1–15. 10.1098/rsif.2010.0270 20829246 PMC3024825

[58] Korolev KS, Xavier JB, Gore J (2014) Turning ecology and evolution against cancer. Nature Reviews Cancer 14, 371–380 10.1038/nrc3712 24739582 PMC13213539

[59] Tabassum DP, Polyak K (2015) Tumorigenesis: it takes a village. Nature Reviews Cancer 15, 473–483 10.1038/nrc3971 26156638

[60] Mitri S.; Clarke E. and Foster K.R. (2015) Resource limitation drives spatial organization in microbial groups. The ISME Journal 10, 1471–1482. 10.1038/ismej.2015.208 26613343 PMC5029182

[61] K. L., Waite A., and Shou W. 2012 Adaptation to a new environment allows cooperators to purge cheaters stochastically. Proc. Natl. Acad. Sci. USA 109, 19079–19086. 10.1073/pnas.1210190109 23091010 PMC3511115

[62] Korolev K.S.; Müller M.J.I.; Karahan N.; Murray A.W.; Hallatschek O. and Nelson D.R. (2012). Selective sweeps in growing microbial colonies. Phys. Biol. 9, 026008 10.1088/1478-3975/9/2/026008 22476106 PMC3359763

[63] Jang Seunghee S., et al Specification and Simulation of Synthetic Multicelled Behaviors. ACS synthetic biology, 2013, vol. 2, no 12, p. 705–714. 23651290 10.1021/sb300034m

[64] Carbonell-Ballestero M; Garcia-Ramallo E; Montañez R; Solé R; Macía J and Rodríguez-Caso C. A bottom-up characterization of transfer functions for synthetic biology designs: lessons from enzymology. Nucl. Acids Res. 2014, 42, 14060–14069. 10.1093/nar/gku964 25404136 PMC4267673

[65] Carbonell-Ballestero M; Duran-Nebreda S; Montañez R; Rodríguez-Caso and Macía, J C. Dealing with the genetic load in bacterial synthetic biology circuits: convergences with the Ohm’s law. Nucl. Acids Res. 2016, 44 (1): 496–507. 10.1093/nar/gkv1280 26656950 PMC4705654

[66] Kashiwagi A; Sakurai T; Tsuru S; Ying BW; Mori K; et al Construction of Escherichia coli gene expression level perturbation collection. Metab Eng 2009, 11, 56–63. 10.1016/j.ymben.2008.08.002 18790072

