# Supplementary figures and images for: Spatial dynamics of synthetic microbial mutualists and their parasites
Source: PLoS Comput Biol. 2017 Aug 21;13(8):e1005689. doi: 10.1371/journal.pcbi.1005689 (PMC5584972; doi:10.1371/journal.pcbi.1005689)

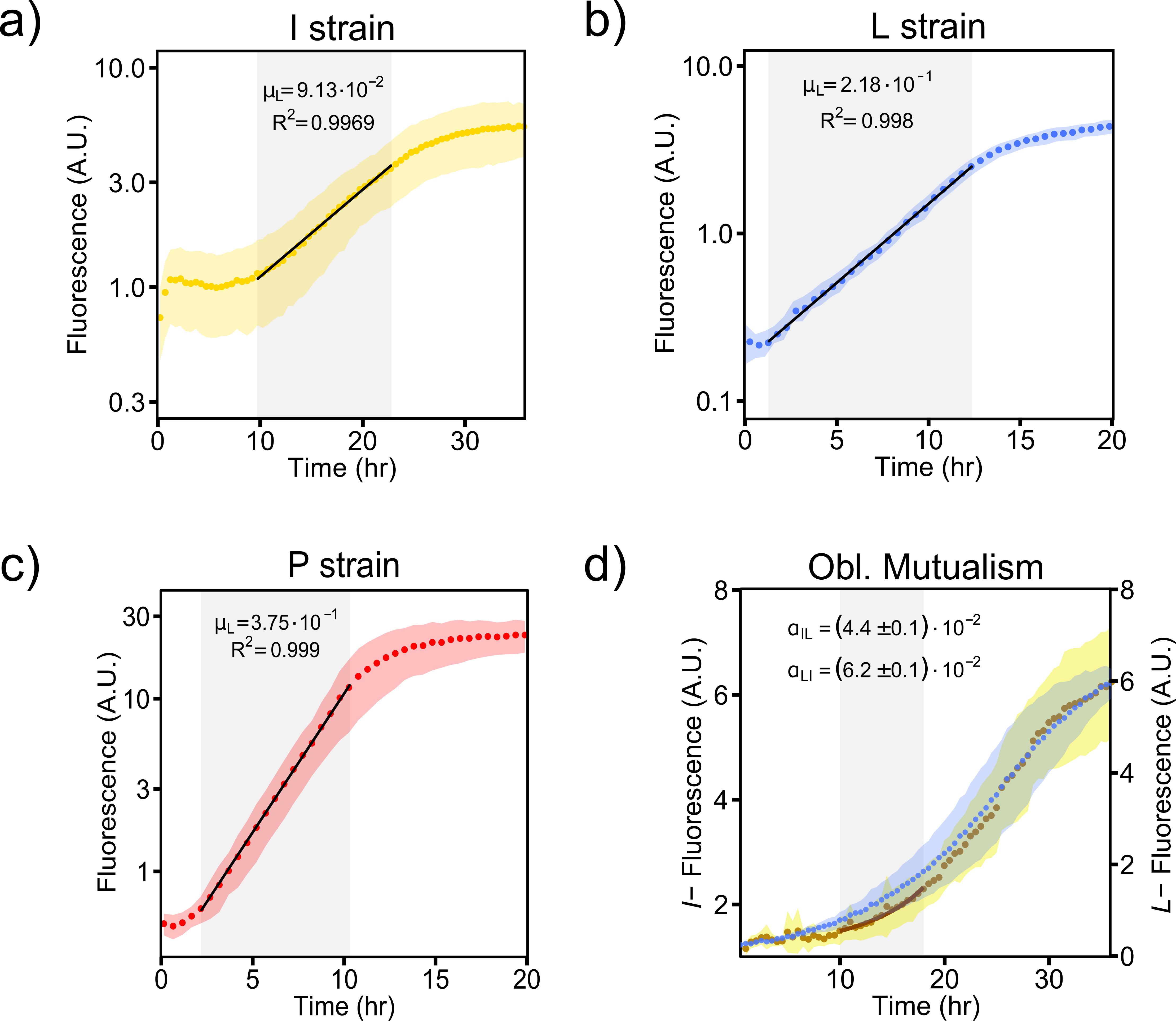

Supplement: S1 Fig — a) Time series for the fluorescence of the I - strain, when cultured in M63 medium supplemented with 100 μM of both iso and leu. Coloured dots stand for the average values across 9 replicates (three technical replicates from each of three biological replicates), shaded area indicates standard deviation. The Malthusian growth rate μI was obtained by linear regression (black solid line) to the data during the exponential growth regime (region delimited by the vertical dashed lines), as described in S1 Text. b) Malthusian growth rate for the L- strain (growth conditions as in a)). c) Malthusian growth rate for the P strain (growth conditions as in a)). Hyperbolic growth rates αIL and αLI were obtained from the observed growth at low population densities (region between dashed lines), as described in S1 Text. The time series correspond to the growth of both I - and L- strains in coculture, in M63 medium with no supplemented amino acids. (TIFF) [file pcbi.1005689.s001.tiff]

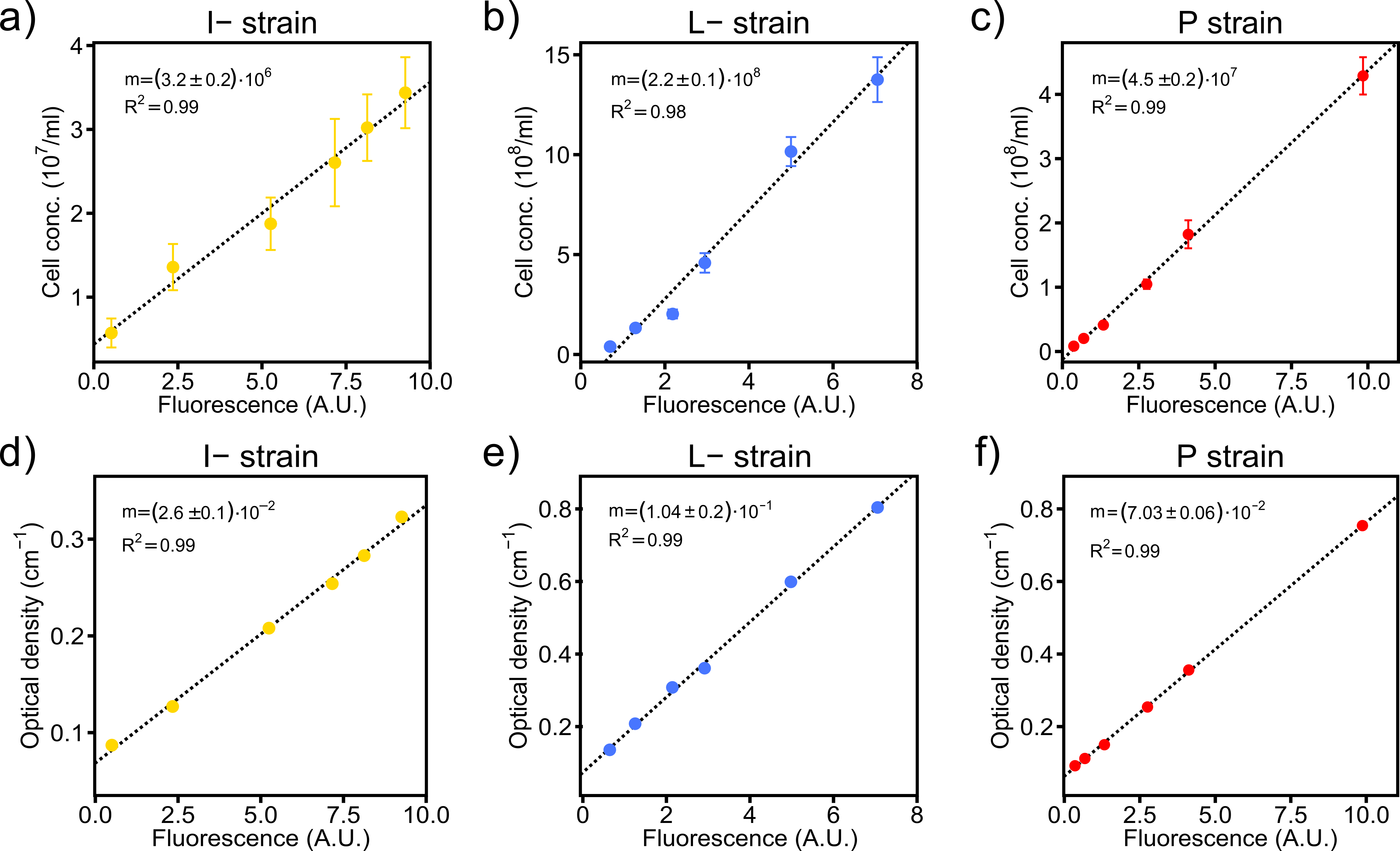

Supplement: S2 Fig — a) Cell concentration in liquid cultures of the I - strain according to their fluorescence. The value of a indicates the slope (in ml−1) obtained by linear regression of the data points. b) In agreement with cell concentration, optical density also scales linearly to fluorescence for the I - strain. c) and d) show the same analysis as in a) and, but for the L- (while e) and f) correspond to analogous results for the P strain). (TIFF) [file pcbi.1005689.s002.tiff]

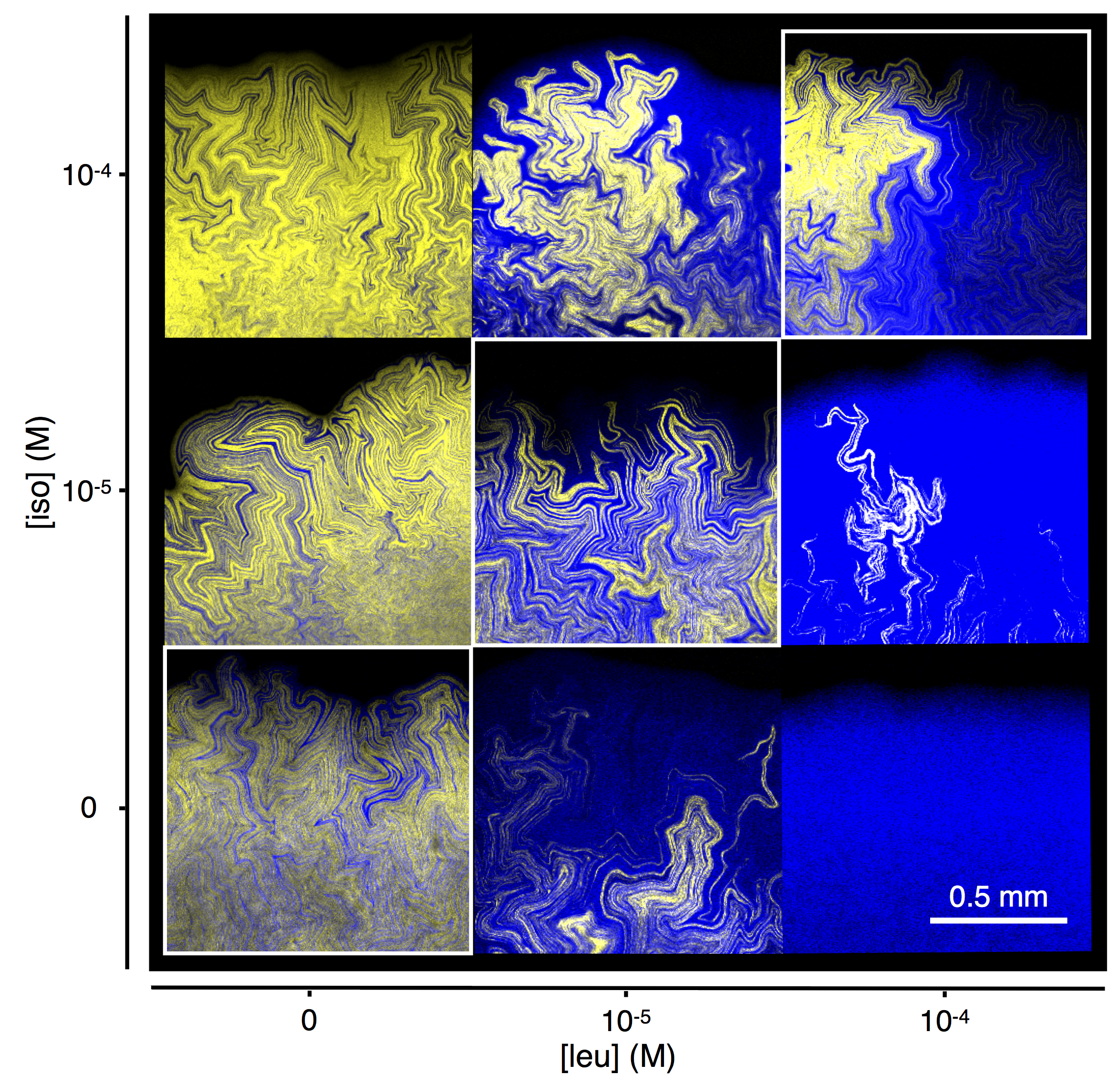

Supplement: S3 Fig — Different concentrations of supplemented iso and leu lead to different spatial dynamics at the edge of the front (e.g., [iso] = 0 and leu = 10−4M leads the L- strain to govern the front). White rectangles indicate the obligate mutualism, facultative mutualism and competition scenarios. (TIFF) [file pcbi.1005689.s003.tiff]

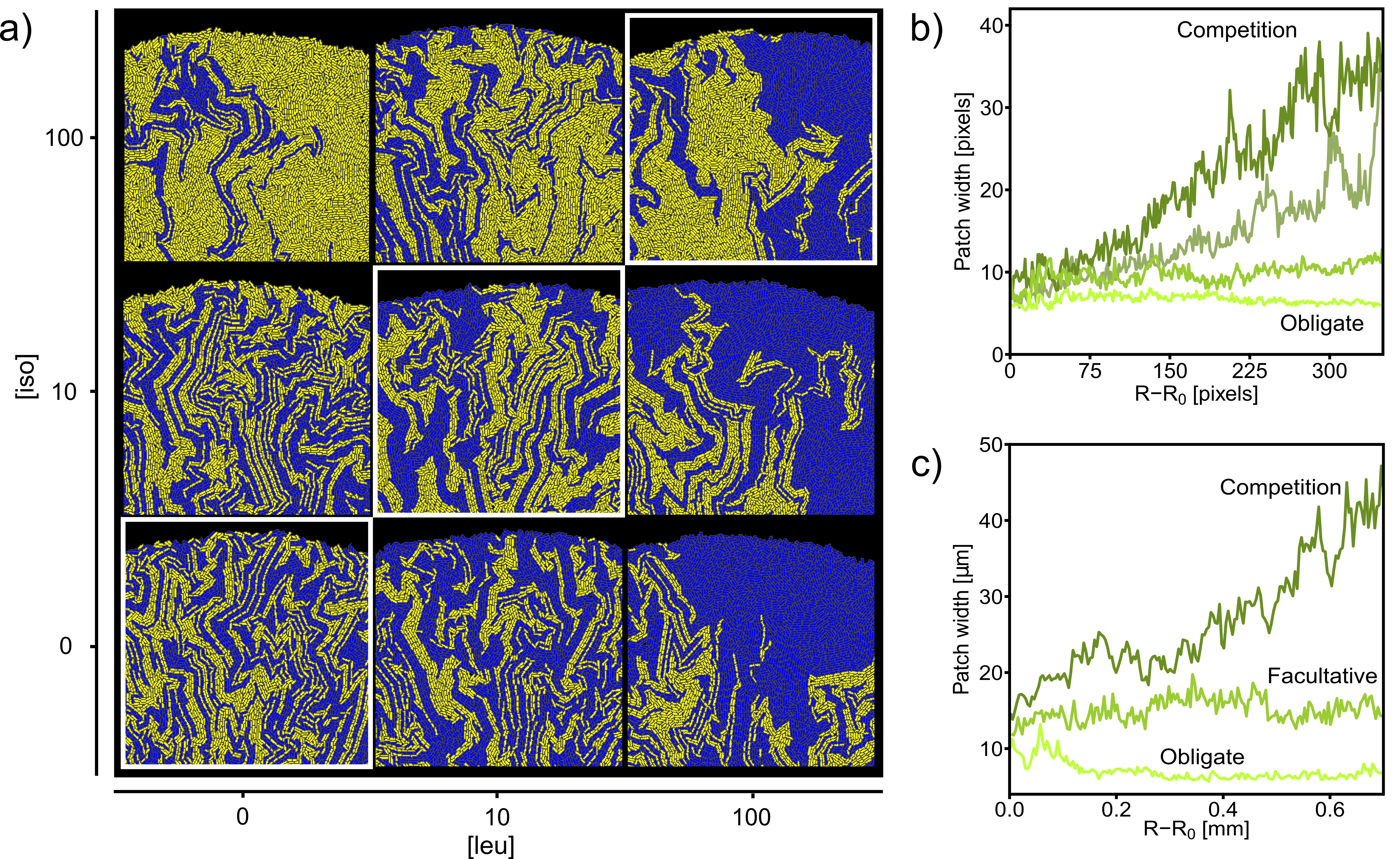

Supplement: S4 Fig — a) Agent-based simulations show analogous scenarios to those observed in Fig 3a. Values on the vertical and horizontal axis indicate the parameter values for the initial extracellular concentration of amino acids (I0 and L0, respectively, see S1 Table). b) Patch width in simulated range expansions, for a different initial extracellular concentration of amino acids (initial nutrient concentration F0 = 90). c) A biological replicate for each of the cases presented in Fig 3c in the Main Text. (TIFF) [file pcbi.1005689.s004.tiff]

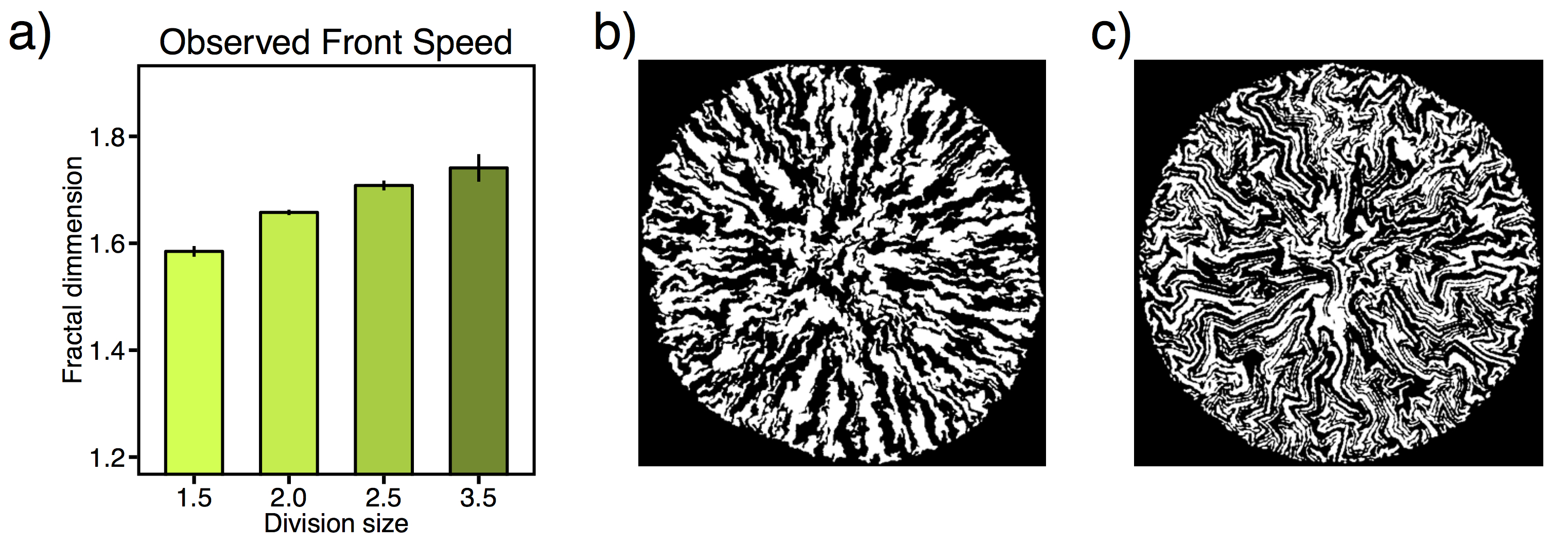

Supplement: S5 Fig — a) Fractal dimension for the boundaries between I - and L- patches in the obligate mutualism scenario. Bars indicate average values, while vertical lines indicate standard deviation from three different simulations. b) A snapshot showing the patches of the I - strain (in white), when de division size parameter is set to 2.0, for a colony with approximately 1.6 × 10^4^ individuals. c) A snapshot showing the patches of the I - strain (in white), when de division size parameter is set to 3.5, for a colony with approximately 1.6 × 10^4^ individuals. (TIFF) [file pcbi.1005689.s005.tiff]

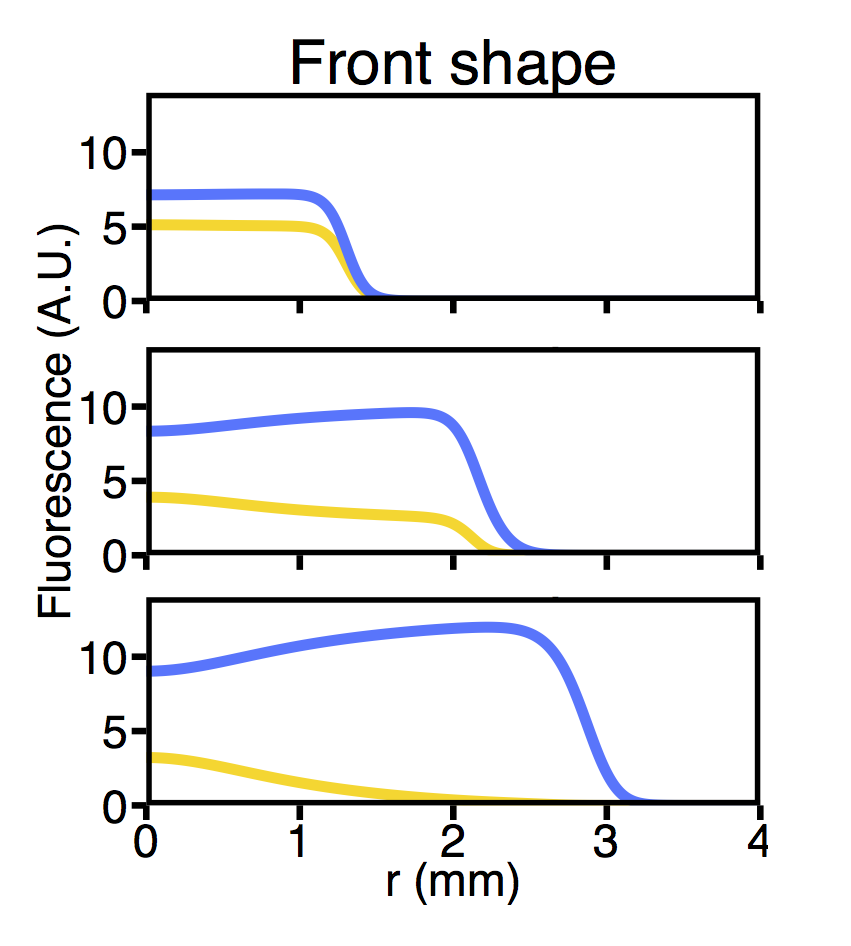

Supplement: S6 Fig — Population density profiles during range expansion of hypercycle strains for different Malthusian growth rates (which models the effect of supplemented amino acids in the medium). The top panel shows the obligate (μi = 0) hypercycle case: the coupled populations propagate as two travelling waves that approximately share the location of their fronts’ edge. In the medium panel (μi = μCi/2), the two species display interactions at the critical intersection that separate mutualism from competition: both strains travel at similar speeds, but the front edge of I - remains slightly behind one of L- due to its smaller growth rate in the presence of amino acids. In the lower panel (μi = μCi), the faster replicator L- wins the competition by conquering the available space long before I -, which is progressively let behind until it is excluded from the population range expansion process. (TIFF) [file pcbi.1005689.s006.tiff]

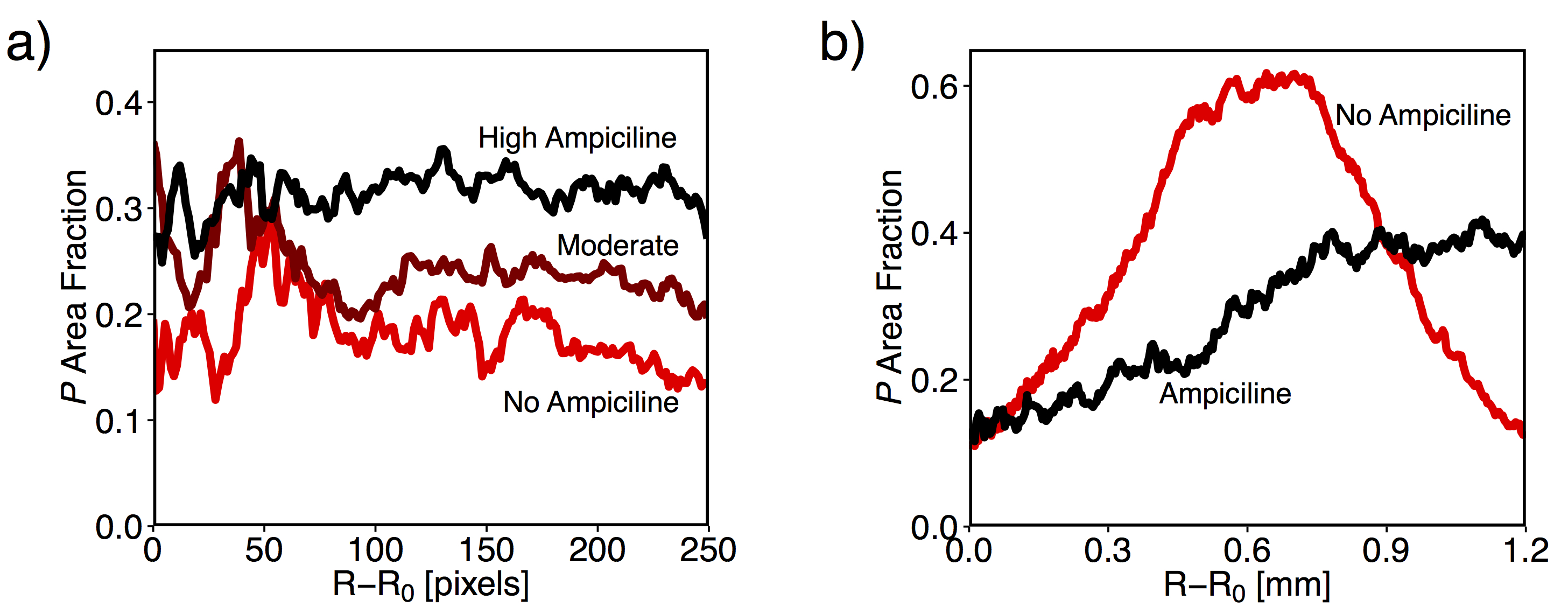

Supplement: S7 Fig — a) In silico, fraction of territory colonized by P cells in three-species population range expansions (curves show average values over 5 simulations). Three different scenarios are shown: no ampicillin (Ampi0 = 0.0, see S1 Table), moderate ampicillin concentration (Ampi0 = 2.0), and high ampicillin concentration (Ampi0 = 4.0). b) Biological replicate for the two scenarios in Fig 4c. (TIFF) [file pcbi.1005689.s007.tiff]

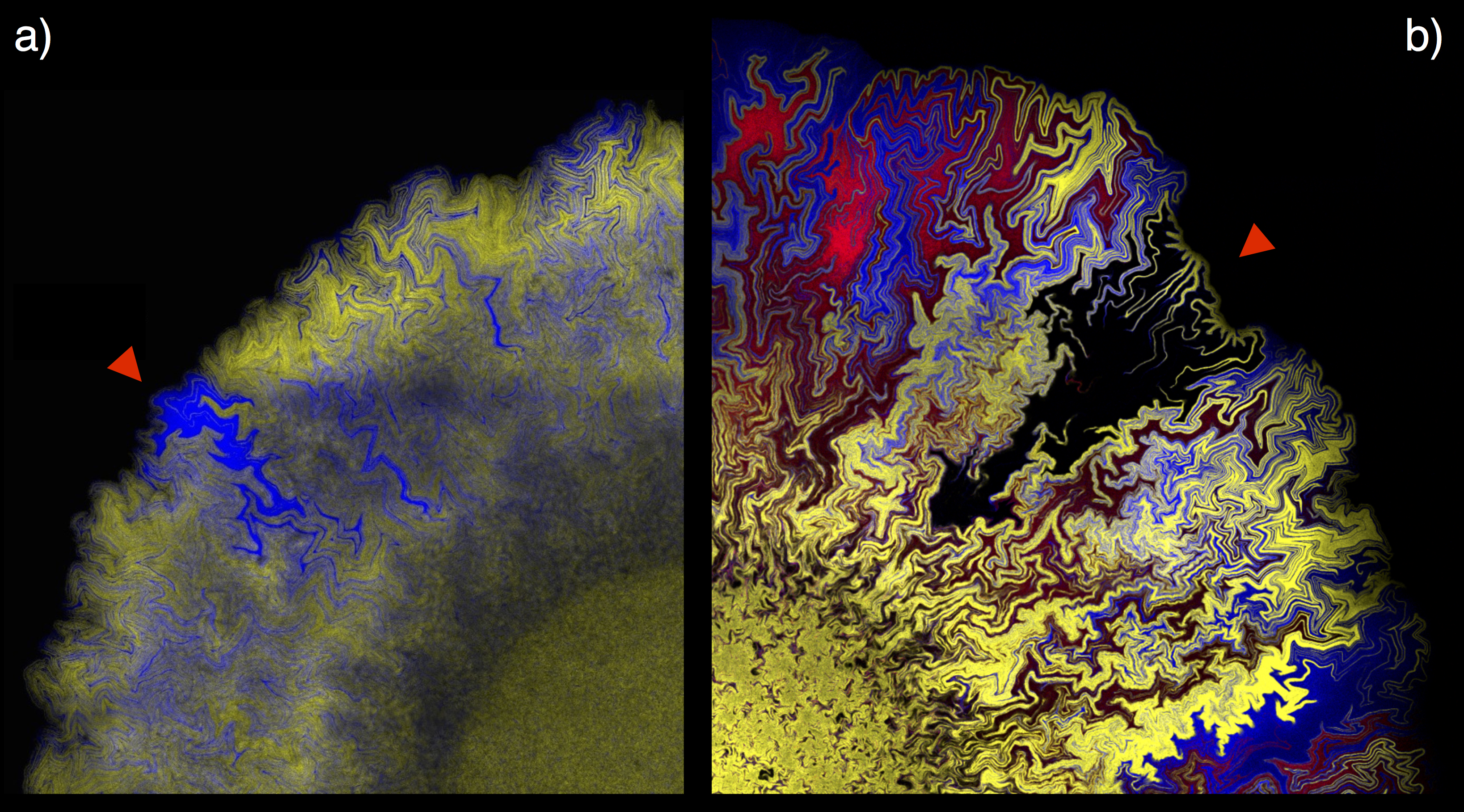

Supplement: S8 Fig — Such mutant sectors were infrequent (less than one mutant sector per colony on average) and were not taken into account for the analysis in the Main text. a) The arrow indicates a mutant sector that reached a significantly wider length than the average length for a L- sector in the colony (obligate mutualism scenario). b) Mutant sector from the P strain exhibiting reduced fluorescent protein expression. (TIFF) [file pcbi.1005689.s008.tiff]
