# Supplementary material for: Spatial dynamics of synthetic microbial mutualists and their parasites
Source: PLoS Comput Biol. 2017 Aug 21;13(8):e1005689. doi: 10.1371/journal.pcbi.1005689 (PMC5584972; doi:10.1371/journal.pcbi.1005689)
Supplement: S1 Table — The table shows the main parameters of the agent-based model, as well as the main processes they affect. Unless stated otherwise in the text, the parameter values used in simulations correspond to those in the source code (S2 Text). (PDF) [file pcbi.1005689.s009.pdf]

**Parameter**	**Descritpion**	**Affects**
F0	Initial extracellular nutrients concentration	Number of active cells and front speed
I0	Initial extracellular isoleucine concentration	Interaction between strains
L0	Initial extracellular leucine concentration	Interaction between strains
Ampi0	Initial extracellular ampiciline concentration	Interaction between strains
Iem	Isoleucine Secretion Rate by L- cells	Hypercycle dynamics
Lem	Leucine Secretion Rate by I- cells	Hypercycle dynamics
Bem	Betalactamase Secretion Rate by P cells	Hypercycle dynamics
Iab	Isoleucine Absorption Rate by I- cells	Hypercycle dynamics
Lab	Leucine Absorption Rate by L- cells	Hypercycle dynamics
Pab	Leucine Absorption Rate by P cells	Parasitism dynamics
Dif	Aminoacids diffusivity	Hypercycle and parasitism dynamics
Fdif	Nutrients diffusivity	Number of active cells and front speed
AmpiDif	Ampiciline Diffusivity	3-species mutualism dynamics
BetaDif	Betalactamase Diffusivity	3-species mutualism dynamics
AmpiDeg	Ampiciline Degradation Rate	3-species mutualism dynamics
BetaDeg	Betalactamase Degradation Rate	3-species mutualism dynamics
kappa	Reaction rate	Ampiciline degradation by Betalactamase
