# Supplementary material for: Spatial dynamics of synthetic microbial mutualists and their parasites
Source: PLoS Comput Biol. 2017 Aug 21;13(8):e1005689. doi: 10.1371/journal.pcbi.1005689 (PMC5584972; doi:10.1371/journal.pcbi.1005689)
Supplement: S1 Text — Approximations for low-density population dynamics used to infer Malthusian and hyperbolic growth rates from experimental data in well-mixed conditions. (PDF) [file pcbi.1005689.s010.pdf]

S1 Text

Growth rates in well-mixed conditions.

In order to measure the Malthusian growth rates associated with competition scenarios, we cultured each of the three strains in M63 medium supplemented with 100 μM of both *iso* and *leu* amino acids. Fluorescence measures showed consistency with the expected exponential growth regime, that precedes growth saturation when the population reaches its carrying capacity, as shown in Fig S1. Malthusian growth rates for the three species were obtained through linear regression of the observed growth data, according to the Malthusian growth model:

$$log(F) = \mu_j t + \beta, \tag{1}$$

where F stands for the fluorescence value, μ_j is the Malthusian growth rate of species j, t stands for the time and β is a constant value for $t = 0$. Thus we obtained the values $\mu_I = (9.1 \pm 0.1) \times 10^{-2}$ hr^{-1}, $\mu_L = (2.18 \pm 0.02) \times 10^{-1}$ hr^{-1} and $\mu_P = (3.75 \pm 0.02) \times 10^{-1}$ hr^{-1}.

In order to obtain an estimate for the hyperbolic growth rates, we considered the well-mixed version of Eq. (2) in the Main text. This correspond to the following set of equations, which do not account for the diffusion process:

$$\begin{aligned} \frac{\partial I}{\partial t} &= \alpha_{IL} IL \left(1 - \frac{I+L}{k}\right), \\ \frac{\partial L}{\partial t} &= \alpha_{LI} IL \left(1 - \frac{I+L}{k}\right), \end{aligned} \tag{2}$$

where we have also assumed that we deal with the obligate mutualism scenario ($\mu_I = \mu_L = 0$).

Considering an approach for the growth at low population densities, it is easy to obtain a solution for $I(t)$ and $L(t)$. Thus, let us neglect the carrying capacity effects [last term on the right hand side of the set of Eqs. (2)], which leads us to:

$$\begin{aligned} \frac{\partial I}{\partial t} &= \alpha_{IL} IL, \\ \frac{\partial L}{\partial t} &= \alpha_{LI} IL \end{aligned} \tag{3}$$

The above set (3) permits to write $I(t)$ in terms of $L(t)$ as:

$$I = \frac{\alpha_{IL}}{\alpha_{LI}} L + \delta, \tag{4}$$

with $\delta = I_0 - \alpha_{IL} L_0 / \alpha_{LI}$ (where I_0 and L_0 stand for the population density numbers at the initial instant t_0). Replacing Eq. (4) into (3), and solving the corresponding differential equation we get the following solution for $L(t)$:

$$L(t) = \frac{\delta}{exp(-\alpha_{LI}\delta(t+\gamma)) - \alpha_{IL}/\alpha_{LI}}, \tag{5}$$

where

$$\gamma = \frac{a}{\alpha_{LI}} ln(L_0) + \frac{b\alpha_{LI}}{\alpha_{IL}} ln\left(\frac{\alpha_{IL} L_0}{\alpha_{LI}} + \delta\right) - t_0, \tag{6}$$

with $a = 1/\delta$ and $b = -a\alpha_{IL}/\alpha_{LI}^2$.

The above Eqs. (4) and (6) constitute a set that we can adjust to the observed growth in well-mixed conditions, with α_{IL} and α_{LI} as the only adjustable parameters. By applying a least squares algorithm to the the fluorescence time series for the growth of the obligate mutualists, we obtained $\alpha_{IL} = (4.4 \pm 0.1) \times 10^{-2}$hr^{-1} and $\alpha_{IL} = (6.2 \pm 0.1) \times 10^{-2}$ hr^{-1}, as Fig S1 shows.
