# Supplementary material for: Spatial dynamics of synthetic microbial mutualists and their parasites
Source: PLoS Comput Biol. 2017 Aug 21;13(8):e1005689. doi: 10.1371/journal.pcbi.1005689 (PMC5584972; doi:10.1371/journal.pcbi.1005689)
Supplement: S2 Text — Source code used to run our simulations in the gro package [63]. (PDF) [file pcbi.1005689.s011.pdf]

```
include gro

set_theme(dark_theme << [ signals := {{0,0,0},{ 0,0,0 },{0,0,0},{ 0,0,0 },{0,0,0}} ] );
set ( "population_max", 30000 );
//%%%%%%%%%%%%%%%%%%%%%%
// PARAMETER VALUES
b:=20;              // Pixel size
Dif:= 0.2;       // Diffusion coefficient of aminoacids
Ddeg:= 0.0;      // Degradation rate of aminoacids
Fdif:= 0.2;        // Nutrient diffusion
Fdeg:= 0.0;        // Nutrient degradation
F0:= 130;          // Initial nutrient concentration in the agar
L0:= 0.0;          // Initial leucine concentration in the agar
I0:= 0.0;          // Initial isoleucine concentration in the agar
Ampi0:= 0.0;       // Initial Ampiciline concentration in the agar
Beta0:= 0.0;       // Initial Betalactamase concentration in the agar
Lem:= 0.015;       // Leucine emission rate by I cells
Iem:= 0.015;       // Isoleucine emission rate by L cells
Bem:= 0.001;         // Betalactamase emission rate by the parasite
Lab:= 0.02;       // Leucine absorption rate
Iab:= 0.02;       // Isoleucine absorption rate
Pab:= 0.02;      //  Leucine absorption rate of parasitic cells
Fab:= 0.02;      // food absorption rate (we assume the same for all species)
AmpiDif:= 0.075;      // Diffusion coefficient of Ampiciline
AmpiDeg:= 0.00001;      // Degradation rate of Ampiciline
BetaDif:= 0.00052;     // Diffusion coefficient of BetaLactamase
BetaDeg:= 0.0001;      // Degradation rate of BetaLactamase
kappa := 0.12;       // Reaction rate at which BetaLactamase degrades Ampiciline
                     // According to this equation: kappa*[Ampi]*[Beta] --> [Beta]

// Region for the initial inoculation
radius:= 15.0;   // determines the size of the initial inoculum of cells
rad:= 0.0;      // rad is only a location to store a random number
omega:= 0.0;   // omega is only a location to store a random nuber
//Counters:
num_cells:= 120;  //  counts total number of cells
num_I:= 40;      // counts the number of I cells
num_L:= 40;      // counts the number of L cells
num_P:= 40;      // counts the number of P cells

//%%%%%%%%%%%%%%%%%%%%%%%
//SETTING UP THE MEDIUM
//Draw the world:
	set ( "signal_element_size", b);
	set ("signal_grid_width", 7200);
	set ("signal_grid_height",7200);
//Signals:
food := signal (Fdif,Fdeg);  // arguments of the function signal: diffusitivy and degradation
iso := signal (Dif, Ddeg);
leu := signal (Dif,Ddeg);
ampi := signal (AmpiDif, AmpiDeg);
beta := signal (BetaDif, BetaDeg);

// REACTIONS IN THE MEDIUM
reaction({ampi,beta},{beta}, kappa); // at rate kappa, the external Betalactamase degrades
Ampiciline
```

```
//%%%%%%%%%%%%%%%%%%%%%
// SYNTHETIC CELLS
//The following comments can be used to change cell morphology and division size
//set ( "ecoli_init_size", 1 );          // size expressed in femtoLiters (fL) units
//set ( "ecoli_division_size_mean", 1.5 ); // fL
//set ( "ecoli_division_size_var", 0.005 ); // fL
program I () := {
    u:=0;
    v:=0;
    w:=0;
    yfp := 50; // I cells are yellow
    daughter : {num_cells := num_cells + 1,
              num_I := num_I + 1
              };   // update cell counting when cells divide
   true : {
     yfp := 75*volume,
     u := get_signal ( food ),      // cells sense external concentrations
     v := get_signal ( iso ),
     w := get_signal (ampi),
   }
   // if there is food then cells absorb food and emit aminoacid
    u > 1 : {   emit_signal (leu, Lem),
              absorb_signal ( food, Fab ),
    }
    // in the absence of Ampi, if there is food and aminoacid, then cells absorb aminoacid and grow
    w < 1 & u > 1 & v > 1 : {  absorb_signal ( iso, Iab ),
                   set ( "ecoli_growth_rate", 0.0346574 )
                  }
    // if there is no aminoacid or no food, cells do not grow
    u < 1 | v < 1 : {set ( "ecoli_growth_rate", 0.0346574 * 0.0 )}
   // also, if Ampiciline is above a threshold, cells do not grow
    w > 1 : {set ( "ecoli_growth_rate", 0.0346574 * 0.0 )}
};

program L () := {
    u:=0;
    v:=0;
    w:=0;
    cfp := 50;   // L cells are blue
    daughter : {num_cells := num_cells + 1,
              num_L := num_L + 1
              };
   true : {
     cfp := 40*volume,
     u := get_signal ( food ),
     v := get_signal (leu),
     w := get_signal (ampi),
   }
   w < 1 & u > 1 : {    emit_signal (iso, Iem),
              absorb_signal ( food, Fab ),
   }
   w < 1 & u > 1 & v > 1 : {  absorb_signal ( leu, Lab ),
                   set ( "ecoli_growth_rate", 0.0346574 )
                 }
```

```
    u < 1 | v < 1 : {set ( "ecoli_growth_rate", 0.0346574 * 0.0 )}
    w > 1 : {set ( "ecoli_growth_rate", 0.0346574 * 0.0 )}
};

program P () := {
     u:=0;
     v:=0;
     w:=0;
     rfp := 50; // parasite cells are red
     daughter : {num_cells := num_cells + 1,
                 num_P := num_P + 1
                };
    true : {
      rfp := 75*volume,
      u := get_signal ( food ),
      v := get_signal (leu),
      w := get_signal (ampi),
    }
    // if there is food, then the parasite absorbes food and emits Betalactamase
    u > 1 : {
              absorb_signal ( food, Fab ),
              emit_signal ( beta, Bem)
    }
    w < 1 & u > 1 & v > 1 : {  absorb_signal ( leu, Lab ),
                   set ( "ecoli_growth_rate", 0.0346574 *1.1 )
                   // the parasite grows 10% faster than I or L
                 }
    u < 1 | v < 1 : {set ( "ecoli_growth_rate", 0.0346574 * 0.0 )}
    w > 1 : {set ( "ecoli_growth_rate", 0.0346574 * 0.0 )}

};

//%%%%%%%%%%%%%%%%%%%%%%%%%%%%%%%%
// THE MAIN LOOP
program main () := {
    r1:= -90;
    r2:= -90;
    t := -10;
    tr:=10;
    t1:=11;
    sim:=0.0;
    // %%%%%%%%%%%%%%%%%%%%%%%%%%%%%%%%%%%%%%%%
    // SOWING THE NUTRIENTS AND INOCULATING CELLS
    //First we sow the borders
    t1 = 11 : {
         set_signal ( food, -90*b, r1*b, F0 ),
         set_signal ( leu, -90*b, r1*b, L0 ),
         set_signal ( iso, -90*b, r1*b, I0 ),
         set_signal ( ampi, -90*b, r1*b, Ampi0 ),
         set_signal ( beta, -90*b, r1*b, Beta0 ),
         set_signal ( food, r1*b, -90*b, F0 ),
         set_signal ( leu, r1*b, -90*b, L0 ),
         set_signal ( iso, r1*b, -90*b, I0 ),
         set_signal ( ampi, r1*b, -90*b, Ampi0 ),
         set_signal ( beta, r1*b, -90*b, Beta0 ),
         set_signal ( food, 89*b, r1*b, F0 ),
```

```
      set_signal ( leu, 89*b, r1*b, L0 ),
      set_signal ( iso, 89*b, r1*b, I0 ),
      set_signal ( ampi, 89*b, r1*b, Ampi0 ),
      set_signal ( beta, 89*b, r1*b, Beta0 ),
      set_signal ( food, r1*b, 89*b, F0 ),
      set_signal ( leu, r1*b, 89*b, L0 ),
      set_signal ( iso, r1*b, 89*b, I0 ),
      set_signal ( ampi, r1*b, 89*b, Ampi0 ),
      set_signal ( beta, r1*b, 89*b, Beta0 ),
      r1 := r1 + 1,
}
// Then we sow inside
t1 = 10 : {
      set_signal ( food, r1*b, r2*b, F0 ),
      set_signal ( leu, r1*b, r2*b, L0 ),
      set_signal ( iso, r1*b, r2*b, I0 ),
      set_signal ( ampi, r1*b, r2*b, Ampi0 ),
      set_signal ( beta, r1*b, r2*b, Beta0 ),
      r1 := r1 + 1,
}
r1 > 89 : {
        r1 := -90,
        r2 := r2 + 1,
}
//Transition from sowing borders to sow inside
t1 = 11 & r2 = (-89) : {
        t1 := 10,
        r2 := -90,
}
r2 > 89 : {
         r2 := -90,
         t1:= -100,
}
// We will let some time to avoid any nutrient gradients
// resulting from diffusion effects during sequential-seeding process
t1 < (-1) : { t1:= t1 + dt }
t1 > (-1) & t1 <  0 : { t1 := 0 }
t1 =0 : {

//%%%%%%%%%%%%%%%%%%%%%%%%%%%%%%%
          // SEEDING INITIAL CELLS
          foreach q in range 40 do {
             set ("rad", rand(radius)),
             set ("omega", 2* 0.01*rand(314)),
             ecoli ( [
                // Seeding cells inside a circle
                x := rad * cos(omega),
                y := rad * sin (omega),
                theta := 0.01*rand(314),
                volume := 1.57 + rand (157) * 0.01 ],
                program L () ),

             set ("rad", rand(radius)),
             set ("omega", 2* 0.01*rand(314)),
             ecoli ([
                // Seeding cells inside a circle
```

```
                    x := rad * cos(omega),
                    y := rad * sin (omega),
                    theta := 0.01*rand(314),
                    volume := 1.57 + rand (157) * 0.01 ],
                    program I () ),

                  set ("rad", rand(radius)),
                  set ("omega", 2* 0.01*rand(314)),
                  ecoli ( [
                    // Seeding cells inside a circle
                    x := rad * cos(omega),
                    y := rad * sin (omega),
                    theta := 0.01*rand(314),
                    volume := 1.57 + rand (157) * 0.01 ],
                    program P () ),
                 }
                 end;
                 start(),
                 t := 0,
                 t1 := 200,
   }

   t > (-1) : {
        t := t + dt,
        tr := tr + dt
     };
   tr >= 20 : {
          snapshot
( "Ampi0_"<>tostring(Ampi0)<>"time"<>tostring(t)<>"numI_"<>tostring(num_I)<>"numL_"<>tostring
(num_L)<>"numP_"<>tostring(num_P)<>".tif" ),
          tr := 0 + dt
   };

t > 900.0  : {stop()};
};
```
