# Supplementary material for: Spatial dynamics of synthetic microbial mutualists and their parasites
Source: PLoS Comput Biol. 2017 Aug 21;13(8):e1005689. doi: 10.1371/journal.pcbi.1005689 (PMC5584972; doi:10.1371/journal.pcbi.1005689)
Supplement: S3 Text — Derivation of the theoretical front speed for one-species hypercycles. (PDF) [file pcbi.1005689.s012.pdf]

S3 Text

Front speed for one-species hypercycles In the Main Text, we have derived an analytical solution for the front speed of two-species hypercycles. For completion, we here present the theoretical speed that would correspond to a one-species hypercycle. Analogous theoretical front speeds for one species mutualistic populations are also described in Refs. [1, 2].

Let's consider an expanding one-species hypercyclic population (u), modelled under the following reaction-diffusion approach:

$$\frac{du}{dt} = D(\frac{\partial^2 u}{\partial x^2}) + ru^2(1-u) \tag{1}$$

We are interested on the invasion speed to the hypercycle. As a first approach, we will consider that the front is planar, and then the one-dimensional speed is a good approximation of the speed of the front in two dimensions. We rescale t and x in order to work with dimensionless variables: $t^* = rt$, and $x^* = x(\frac{r}{D})^{1/2}$.

Then, Eq. (1) becomes:

$$\frac{du}{dt^*} = \frac{\partial^2 u}{\partial x^{*2}} + u^2(1-u) \tag{2}$$

We look for front propagation solutions, so let us assume that there exist solutions to Eq. (2) with the propagating wave form:

$$u(x^*, t^*) = U(z) = \frac{1}{(1+ae^{bz})^s}, \tag{3}$$

with $b, s > 0$ and $z = x^* - ct*$. Using, $u_x = U_z = U'$, and $u_t = -cU_z = cU'$ (where the subscripts denote the corresponding partial derivatives), we obtain the expressions for the following partial derivatives:

$$u_x = -sabe^{bz}(1+ae^{bz})^{-s-1} \tag{4}$$

$$u_{xx} = s(s+1)a^2b^2e^{2bz}(1+ae^{bz})^{-s-2} - sab^2e^{bz}(1+ae^{bz})^{-s-1} \tag{5}$$

$$u_t = csabe^{bz}(1+ae^{bz})^{-s-1} \tag{6}$$

We rewrite Eq. (2) as:

$$U'' + cU' + u^2(1-U) = 0, \quad \forall z \tag{7}$$

Rewriting Eq. (7), and reorganising terms as powers of e^{bz} we obtain:

$$\begin{aligned} e^{2bz}(s(s+1)a^2b^2 - sa^2b^2 - csa^2b) \\ +e^{bz}(-sab^2 - csab) \\ +(1+ae^{bz})^{-s+2} - (1+ae^{bz})^{-2s+2} = 0 \end{aligned} \tag{8}$$

The above Eq. (8) has to be equal to zero $\forall z$. Then ,the coefficients of e^0, e^bz, and e^2bz must all be identical to zero. Taking into account that we look for travelling waves solution of the form (7) (with $s > 0$), it is easy to show that the only value that s can take is $s = 1$. Using this into Eq. (7) leads to:

$$\begin{aligned} e^{2bz}(a^2b^2 - ca^2b) \\ +e^{bz}(-ab^2 - cab + a) = 0, \end{aligned} \tag{9}$$

which leads to the dimensionless front speed:

$$c = b = \sqrt{1/2} \tag{10}$$

Recovering dimension variables from the front speed c we obtain: which leads to the dimensionless front speed:

$$v = \sqrt{rD/2.} \tag{11}$$

References

1. Murray, James D. Mathematical Biology I: An Introduction. Springer, Berlin, 2004.
2. Korolev, Kirill S. (2013). The fate of cooperation during range expansions. PLOS Comp Biol 9, e1002994.
